# Ultrasensitive, Specific, and Rapid Fluorescence Turn‐On Nitrite Sensor Enabled by Precisely Modulated Fluorophore Binding

**DOI:** 10.1002/advs.202002991

**Published:** 2020-11-13

**Authors:** Zhiwei Ma, Jiguang Li, Xiaoyun Hu, Zhenzhen Cai, Xincun Dou

**Affiliations:** ^1^ Xinjiang Key Laboratory of Explosives Safety Science Xinjiang Technical Institute of Physics & Chemistry Key Laboratory of Functional Materials and Devices for Special Environments Chinese Academy of Sciences Urumqi 830011 China; ^2^ Center of Materials Science and Optoelectronics Engineering University of Chinese Academy of Sciences Beijing 100049 China

**Keywords:** benzothiazole, binding site, fluorescence turn‐on, nitrite detection, portable sensing

## Abstract

The precise regulation of fluorophore binding sites in an organic probe is of great significance toward the design of fluorescent sensing materials with specific functions. In this study, a probe with specific fluorescence properties and nitrite detection ability is designed by precisely modulating benzothiazole binding sites. Only the fluorophore bond at the *ortho*‐position of the aniline moiety can specifically recognize nitrite, which ensures that the reaction products displays a robust green emission. The unique 2‐(2‐amino‐4‐carboxyphenyl) benzothiazole (*ortho*‐BT) shows superior nitrite detection performance, including a low detection limit (2.2 fg), rapid detection time (<5 s), and excellent specificity even in the presence of >40 types of strong redox active, colored substances, nitro compounds, and metal ions. Moreover, the probe is highly applicable for the rapid on‐site and semiquantitative measurement of nitrite. The proposed probe design strategy is expected to start a new frontier for the exploration of probe design methodology.

## Graphical abstract

The regulation of the benzothiazole (BT) binding sites, including the *para*‐, *meso*‐, and *ortho*‐positions on the aniline moiety is found to be an efficient strategy to facilitate a fluorescent turn‐on of *ortho*‐BT toward nitrite. The proposed strategy is expected to pave a brand‐new way for the specific detection of nitrite and shine light on the development of probe design methodology.

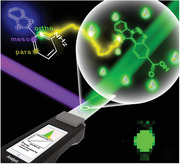

## 1 Introduction

The capability to detect and identify toxic and harmful substances precisely, sensitively, and rapidly is of great importance since it can effectively prevent serious threats or hazards to public health arising from industrial pollution or major emergencies. To realize the detection and identification of hazardous analytes, numerous efforts have been undertaken in the fields of colorimetry, fluorescence, and Raman spectroscopy, and gas sensing to explore pioneering methodologies over the past few decades.^[^ ^1^ ^]^ Among them, organic fluorescent probes, which have the advantages of adjustable structures, variety of functional groups, high luminescence efficiency, fast response, and specific recognition sites have attracted considerable attention^[^ ^2^ ^]^ and serve as an effective tool in bioimaging,^[^ ^3^ ^]^ ion analysis,^[^ ^4^ ^]^ explosive detection,^[^ ^5^ ^]^ gas sensing,^[^ ^6^ ^]^ etc. Moreover, various studies have reported that fluorophores, such as BODIPY, coumarin, pyronin, and benzothiazole, can be used as a fluorophore toward the design of high performance probes.^[^ ^1^, ^7^ ^]^ Generally, significant attention has been devoted to modifying the recognition group in the fluorophore and regulating the energy transfer process to realize the efficient analyte detection. Some modification techniques include tuning the photophysical properties of aza‐BODIPY dyes by changing the peripheral substituents,^[^ ^8^ ^]^ modifying the boronate ester moiety on the fluorophore via chemoselective phenylboronate–phenol transformation,^[^ ^9^ ^]^ and adjusting the photoinduced electron transfer process toward nitrite under acidic conditions upon reaction with an amino group bonded on the organic fluorophore.^[^ ^10^ ^]^ From the perspective of the chemical structure of these probes, the binding sites of the fluorophore are of vital importance toward regulating the optical properties and their resulting detection performance. Therefore, it is of great importance to design a specific functional probe by the precise regulation of the fluorophore binding site. However, exploration of this crucially important methodology has been ignored for a long time and has moved with unusually low speed over the long history of fluorescence detection.

Nitrite as a toxic and harmful substance is widely used in processed food products, biomedicines, and chemicals because of its colouring, antiseptic, and antioxidant properties.^[^ ^11^ ^]^ However, nitrite can react with an imine under acidic conditions to produce nitrosamine carcinogens^[^ ^12^ ^]^ and can cause the oxidation of normal oxygen‐carrying haemoglobin leading to their irreversible conversion into methaemoglobin, which seriously endangers human health.^[^ ^11^, ^13^ ^]^ Furthermore, nitrite can easily be oxidized to nitrate and be further used as an important raw material to make improvised explosive devices (IEDs), causing a serious threat to world safety.^[^ ^14^ ^]^ To achieve the precise, sensitive, and rapid detection of trace amounts of nitrite, the most commonly adopted method employs an amino or aniline group to decorate the designated luminophore. However, this method finds it difficult to distinguish nitro compounds from nitrite. In addition, the resulting diazonium salt can easily decompose and the response time usually spans from half an hour to several hours. Therefore, it will be of great significance if the urgent need for a highly sensitive, anti‐interfering, rapid, and semiquantitative method of identifying nitrite can be realized by developing a new detection strategy based on adjusting the fluorophore binding site. This is also expected to provide firm evidence for the crucial importance of adjusting the binding sites to modulate the detection performance.

To realize the sensitive, specific, rapid qualitative, and semiquantitative fluorescence detection and recognition of nitrite, the design of a responsive probe is expected to meet the following criteria: (i) Specific recognition sites toward nitrite together with low background noise enabled specific sensing to exclude the influence from interferents; (ii) Remarkable photochemical/physical properties change or special response mode enabled sensitive, large response range, and rapid detection toward the target analyte; (iii) Stable fluorescence detection performance at different pH values since the introduction of nitrite analyte may greatly influence the pH value.

To this end, we report the regulation of the binding sites, including the *para*‐, *meso*‐, and *ortho*‐positions of benzothiazole on the aniline moiety (*para*‐BT, *meso*‐BT, and *ortho*‐BT) and the resulting ultrasensitive, specific and rapid sensing performance toward nitrite. A new detection mechanism for nitrite with a fluorescence turn‐on mode of action has been discovered on the basis of the reaction of *ortho*‐BT toward nitrite. The specific reaction enables the original fluorescence emission to change from none to green (530 nm) within 5 s, and the detection limit could reach 40 × 10^−9^ m and 2.2 fg. In addition, it has been proven that the presence or absence of >40 types of oxidative or reductive substances at one thousand times higher concentration has a negligible effect on the detection performance. The proposed fluorescent probe design strategy was further proven to be instrumentally applicable for the rapid, on‐site, and semiquantitative measurement of nitrite. We expect this tentative study will pave a brand‐new way for the specific sensor and shine light on the development of probe design methodology.

## 2 Results and Discussion

### 2.1 Precisely Modulated Binding of Benzothiazole on Aniline Moiety

**Figure 1:**
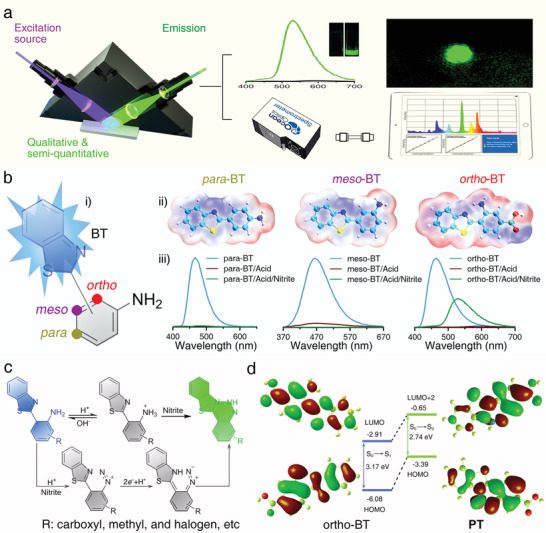
a) Schematic representation of the fluorescence turn‐on mode of action and the portable strategy for the detection and discrimination of nitrite. b) The Schematic illustration of (i) the regulation of the fluorophore binding at the *para*‐, *meso*‐, and *ortho*‐positions of the aniline moiety, and (ii) the corresponding maps of the electrostatic potential surfaces of *para*‐BT, *meso*‐BT, and *ortho*‐BT, and (iii) the corresponding fluorescence spectra of the probes, probes under acidic conditions, and probes after the detection of nitrite, respectively. c) Proposed detection mechanism of *ortho*‐BT toward nitrite. d) Calculated electron‐density distribution and corresponding energy diagrams of the main molecular orbitals of *ortho*‐BT and the reaction products.

The specific fluorescence turn‐on mode of action of the sensor can not only resolve the issue of the qualitative analysis of analyte, but also realize the semi‐quantitative analysis of analytes by optical signals with help from the chemometric method. To achieve the qualitative and semiquantitative features of fluorescence sensor, a portable detection platform was built, and a probe with excellent sensing properties was depicted and designed (**Figure** **1**a). Benzothiazole is a fluorophore bearing a thiazole group fused on a benzene ring, which forms a large *π* bond in the conjugated rigid plane, allowing for the formation of the organic probe, through the combination of benzothiazole and an aniline moiety bearing a nitrite recognition site, having excellent photothermal stability and fluorescence stability. Based on the excited‐state intramolecular proton‐transfer (ESIPT) in 2‐(amino‐phenyl) benzothiazole, an extremely fast phototautomerization event could take place. Upon photoexcitation, the molecule electronic charge can be redistributed, and there is a structural tautomerism between =N— and —NH— on the thiazole heterocycle together with NH_2_— and NH= on the aniline moiety.^[^ ^15^ ^]^ Because benzothiazole has an adjustable binding site on the aniline moiety (Figure 1b(i)), probes with the fluorophore at the *para*‐, *meso*‐, and *ortho*‐positions of the aniline moiety were prepared and characterized through NMR and mass spectroscopy (Figures S1–S9, Supporting Information). Natural bond orbital methods were used to investigate the electrostatic potential surface (EPS) of probes to evaluate their preferential reaction sites for nitrite (Figure 1b(ii)). It is observed that the EPS obtained for all the probe amines were positive and the maximum value was located near the N–H bond (red region), indicating the strong attraction in this region to nitrite. It is worth noting that when benzothiazole was functionalized at the *ortho*‐position of the aniline moiety, the EPS of the amino group decreases, while that of =N— on the thiazole ring increases simultaneously, implying the possible synergistic effect between the amino group and =N— on nitrite. The fluorescence properties of the probes and the reaction products were further investigated and the effects of the *para*‐, *meso*‐, and *ortho*‐binding sites on the reaction were confirmed. Upon comparison, there was no distinct difference in the peak position (centred at 463 nm with a blue colour) or intensity among the different emissions for *para*‐, *meso*‐, and *ortho*‐BT (Figure 1b(iii)). Upon the introduction of an acidic solution, the fluorescence of all the probes was quenched (Figure S19, Supporting Information). After the addition of nitrite, only the fluorescence of *ortho*‐BT was turned on and an obvious green emission peak appeared at 530 nm. In addition, the absorption peak also shows a homologous red‐shift during this process (Figure S20, Supporting Information), further indicating the formation of a new molecular structure. Whereas there is no sign showing that the *meso*‐BT and *para*‐BT were turned on upon the addition of nitrite.

It is considered that the highly active site (=N—) and diazo group can undergo an intramolecular coupling reaction to form a heterocyclic product (Figure 1c). From the ^1^H NMR spectra, the chemical shift at 7.0 ppm corresponding to the amino proton of the probe disappeared and a new proton signal attributed to the imine appeared at 7.51 ppm (Figures S7–S12, Supporting Information, R: carboxyl). The MS data showed that after the addition of nitrite, the peak at *m*/*z* 270.0 corresponding to *ortho*‐BT disappeared and a new peak at *m*/*z* 283.3 corresponding to reaction products (**PT**) appeared (Figures S9, S12, and S20d, Supporting Information). Meanwhile, upon comparing the integration of the proton signals, the number of spectral splitting peaks, and molecular weight, the formation of **PT** and the proposed reaction mechanism could be confirmed. Thus, it is proved that the amino group on *ortho*‐BT could react with nitrite under acidic condition to form diazo group. Due to the ESIPT in *ortho*‐BT, there is a =N— and —NH— structural tautomerism on the thiazole heterocycle, the diazo group is conjugated and can undergo an intramolecular coupling reaction with the highly active site (—NH—) to form a heterocyclic benzothiazole triazine product, which resulted in the fluorescence turn on sensing mode. Theoretically, the calculated transition energy was 3.17 eV (391.5 nm) for *ortho*‐BT and this value changed to 2.74 eV (459.8 nm) for **PT** (Supporting Information). These results were in good agreement with the experimental data (3.16 and 2.70 eV, respectively), indicating that the new fluorescence emission is attributable to benzothiazole triazine (Figure 1d; Table S1, Supporting Information).

It should be noted that the carboxyl group can be replaced by a methyl or halogen, maintaining the above results (Figures S13–S18 and S21, Supporting Information), which indicates that the *ortho*‐position binding site design leads to the effective generation of the fluorescence signal toward nitrite.

### 2.2 Fluorescent Detection and Discrimination of Nitrite

**Figure 2:**
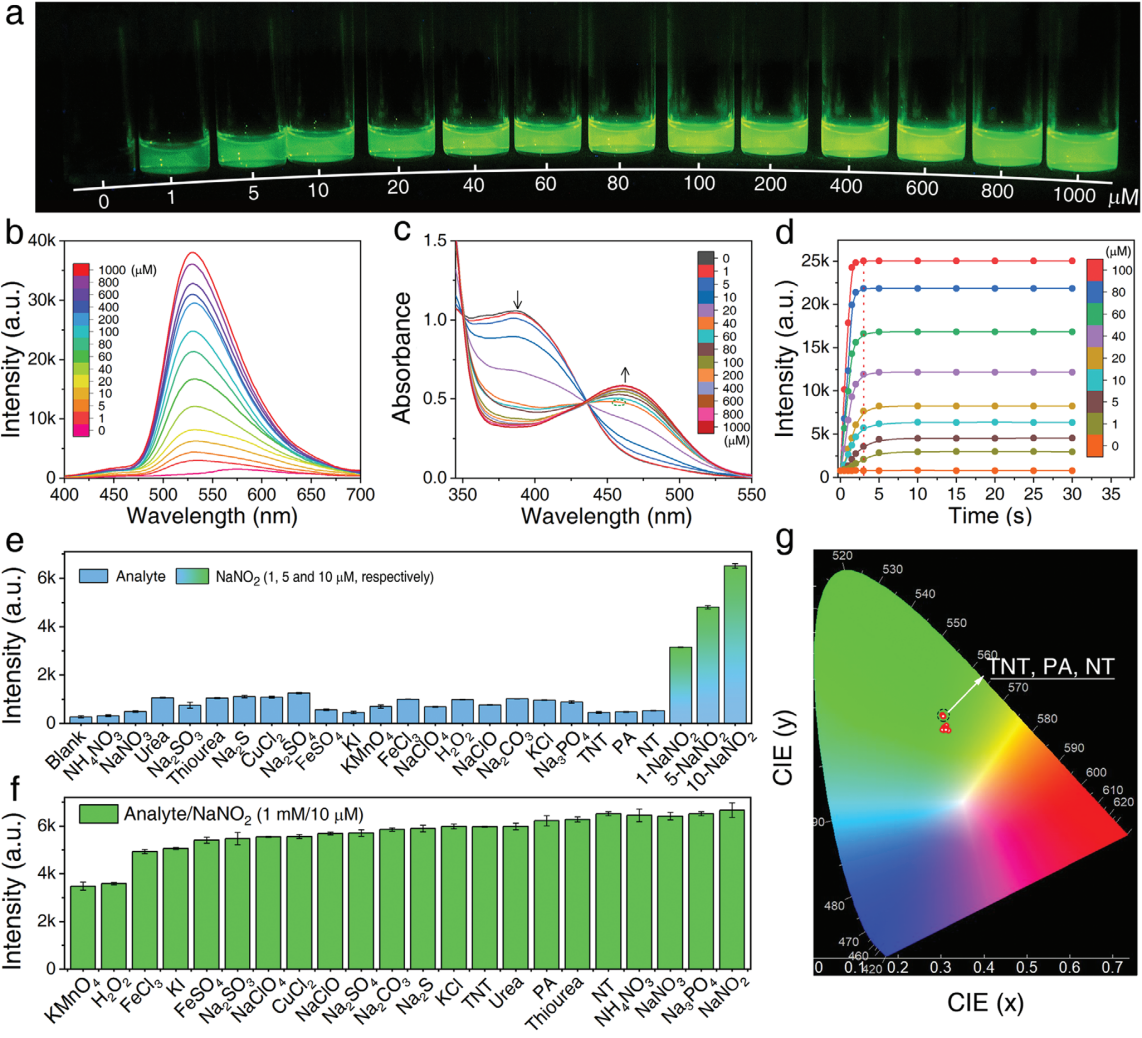
a) Optical images obtained under irradiation with a 365 nm UV lamp. b) Fluorescence spectra and c) UV–vis absorbance spectra obtained for *ortho*‐BT in response to different concentrations of nitrite (0–1000 × 10^−6^ m). d) Time‐dependent fluorescence response of *ortho*‐BT upon the addition of different amounts of nitrite (0–100 × 10^−6^ m). (*λ*_ex_ = 365 nm; *λ*_em_= 530 nm; Slits: 2 nm; Integral time: 100 ms; Spectrometer: Maya 2000pro). Fluorescence intensity changes obtained for *ortho*‐BT in response to e) various analytes (1 × 10^−3^ m), different concentrations of nitrite (1, 5, and 10 × 10^−6^ m), and f) a mixture of nitrite (10 × 10^−6^ m) and various analytes (1 × 10^−3^ m). g) CIE spectral consistency of the corresponding fluorescence shown in inset (f); one point represents the colour coordinates in the presence of nitro interferents, while the other one represents the colour coordinates in the presence of the other interferents. (*λ*_ex_= 365 nm; Slits: 1 nm; *λ*_em_= 530 nm; Slits: 0.3 nm; Integral time: 200 ms; Spectrometer and CIE: Edinburgh FLS1000).

**Figure** **2**a shows the fluorescent images of *ortho*‐BT toward nitrite at concentrations from 0 to 1 × 10^−3^ m under irradiation with a 365 nm UV lamp, in which the green emission increases from none to give a sharp signal. In particular, 1 × 10^−6^ m nitrite can be completely distinguished from that observed in the absence of nitrite. Upon increasing the concentration of nitrite, the fluorescence intensity increases significantly and the colour shows no obvious difference. Furthermore, all the samples were further investigated using fluorescence and UV–vis absorption spectroscopy. Under acidic conditions, the fluorescence of *ortho*‐BT was turned on upon the addition of nitrite with a peak observed at 530 nm whose intensity gradually increased with an increase in the concentration of nitrite (Figure 2b). The origin of the appearance of the green emission was investigated through the absorption spectra of *ortho*‐BT, which show a peak at 388 nm whose intensity gradually decreases upon the addition of nitrite. At the same time, a new peak centred at 460 nm was observed, which becomes obvious when the nitrite concentration reaches 40 × 10^−6^ m (Figure 2c). The increase in the intensity of this new peak gradually decreases and shows no further visible increase when the concentration exceeds 100 × 10^−6^ m, indicating the successful conversion from *ortho*‐BT to **PT**. This change also corresponds to the shift in the energy bandgap observed from 2.69 eV (*ortho*‐BT) to 2.34 eV (**PT**), which confirmed that the new green emission was caused by the narrower bandgap of **PT** (Figure S22a, Supporting Information).

The time‐dependent fluorescence intensities observed at 530 nm were recorded to evaluate the response time and stability toward nitrite (Figure 2d). The fluorescence intensity increases rapidly to 80% of the maximum value within 3 s for all concentrations of nitrite studied and reaches saturation after 5 s, which remains unchanged afterwards (Figure 2d, red dashed line). When compared to the other fluorescence detection methods previously reported (Table S2, Supporting Information), the present *ortho*‐BT exhibits an obvious advantage with rapid and stable characteristics. The rapid detection process is derived from the photochemical process of ESIPT displayed by *ortho*‐BT. This mechanism is specifically due to the rapid transformation of the active sites from an amino to imine and =N— to —NH—, which improves the reactivity of the probe.^[^ ^15^ ^]^ The detection parameters, such as the sensitivity, response rate, and stability of *ortho*‐BT toward nitrite are of significant importance to evaluate the limit of detection (LOD), detection efficiency, and possible practical application. From the linear fitting of the fluorescence intensity, the LOD toward nitrite was determined to be as low as 40 × 10^−9^ m (Figure S22b, Supporting Information), which is much lower than the maximum limit of nitrite (65 × 10^−6^ m) stipulated by the World Health Organization,^[^ ^16^ ^]^ indicating the superior performance of *ortho*‐BT when compared to other nitrite fluorescent detection methods.

To confirm the specificity of *ortho*‐BT toward the detection of nitrite, more than 40 substances consisting of oxidants, reductants, metal ions, and structural analogues were selected as interferents. Upon the addition of the various analytes (1 × 10^−3^ m), the fluorescence spectra show no obvious changes (Figures S23 and S24, Supporting Information). However, the specific fluorescent peak (530 nm) of *ortho*‐BT emerged significantly and caused a noticeable intensity enhancement after adding 1, 5, and 10 × 10^−6^ m nitrite, respectively. More importantly, nitro chemicals (trinitrotoluene, picric acid, and nitromethane, namely TNT, PA, and NT) did not affect the detection of nitrite. Using a statistical comparison of the fluorescence intensity, it can be concluded that *ortho*‐BT has a specific response and stability in the presence of various analytes (Figure 2e).

To further investigate the effect of interferents on the selectivity of *ortho*‐BT, the fluorescence performance was investigated by mixing 10 × 10^−6^ m of nitrite with more than 40 types of analytes (1 × 10^−3^ m). It was observed that the characteristic fluorescence of *ortho*‐BT was turned‐on due to the presence of nitrite and the intensity was maintained at >75% in the presence of all the interferents except for KMnO_4_ or H_2_O_2_, which have a fluorescence quenching effect of up to 52% of the original intensity (Figure 2f; Figures S25 and S26, Supporting Information). It is considered that strong oxidizing substances like H_2_O_2_ at a concentration 100 times higher than that of nitrite may oxidize nitrite to be nitrate, leading to the decrease of the nitrite concentration and thus the fluorescence intensity. For the mixture of KMnO_4_ and nitrite or K_2_Cr_2_O_7_ and nitrite with a pH value close to 7, KMnO_4_ and K_2_Cr_2_O_7_ cannot oxidize nitrite into nitrate. Through comparison of K_2_Cr_2_O_7_ and KMnO_4_ to the detection performance toward nitrite, it is found that KMnO_4_ could absorb the emission of **PT**, which is the key factor inducing the decrease of the fluorescence intensity. To further prove this, we selected a red ink, the absorption of which is similar to KMnO_4_ (Figure S27a, Supporting Information), as a reference. It is shown that the addition of this red ink could also induce a decrease in the fluorescence intensity (Figure S27b,c, Supporting Information). Thus, interferents such as some oxidizing or coloured substances, might absorb the emission of **PT** and induce the fluorescence intensity decrease, but the specific fluorescence turn‐on phenomenon toward nitrite could be observed without obvious influence, indicating the perfect anti‐interference ability of the probe. Moreover, it can be observed from the colour gamut that the exact positions of the spectral coordinates of these interferents strongly overlap with that of nitrite (Figure 2g), except for a little shift resulted from the further quenching of excessive *ortho*‐BT by the nitro compounds.

### 2.3 *Ortho*‐BT‐Embedded Paper‐Based Sensor for Nitrite Solution and Solid Detection

**Figure 3:**
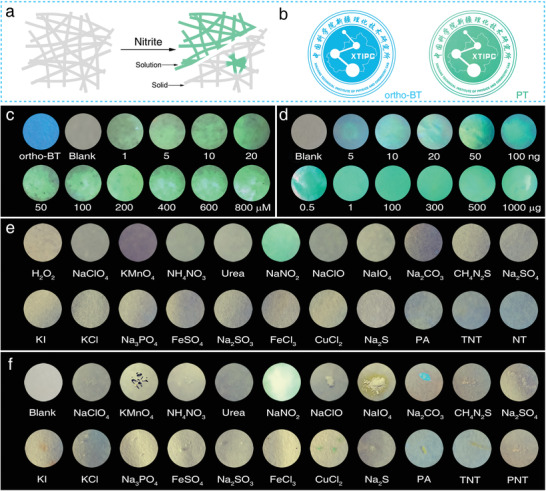
a) Schematic of the test strips used to detect a nitrite solution and solid particles. b) Optical images of the special logo stamped by *ortho*‐BT and **PT**. Optical images of the test strips used to detect c) a nitrite solution (0–800 × 10^−6^ m) and d) nitrite solid (0–1 mg). Optical images of the anti‐interference characterization of the test strips to >20 types of substances in the e) solution and f) solid phase, respectively. All images were obtained under irradiation with a 365 nm UV lamp and all the image diameters in (c)–(f) were maintained at 10 mm. (Excitation source: 365 nm LED; Exposure time: 1/40 s).

To develop a simple, cheap, fast, and reliable sensor toward nitrite detection,^[^ ^5^, ^17^ ^]^ the *ortho*‐BT probe was integrated on filter paper together with acid to obtain a test strip with no fluorescence emission for the detection of a nitrite solution or solid (**Figure** **3**a). By comparing the morphology of the filter paper and corresponding energy dispersive spectroscopy (EDS) mapping results (Figure S28a–c, Supporting Information), it is observed that *ortho*‐BT was uniformly distributed in the filter paper despite the remarkable difference in the microstructure (Figure S28d, Supporting Information). It is considered that the fibrous and pore structure in the fibre provides sufficient space for *ortho*‐BT to come in contact and be uniformly dispersed. The test strip with no fluorescence emission shows a strong and uniform green emission upon contact with the nitrite solution (Figure S28e,f, Supporting Information). Representatively, the patterns of the logo of our institute using *ortho*‐BT and **PT** on filter paper shows blue and green emission with perfect shape retention, demonstrating the reliability of the test strip strategy for restricting the probe and the reaction products in specific areas with no dispersion (Figure 3b).

The detection performance of the test strips toward a nitrite solution and solid were investigated. The test strip shows no emission due to acid induced quenching, forming a sharp contrast with the original strong blue emission of the *ortho*‐BT embedded filter paper (Figure 3c). Upon dropping a nitrite solution (1–800 × 10^−6^ m) onto the test strips, the solution rapidly diffuses and a green emission can be observed within 5 s under irradiation with a 365 nm UV lamp and the intensity gradually increased upon increasing concentration of nitrite. Notably, the test strip can completely discriminate a nitrite solution with a concentration as low as 1000 × 10^−9^ m using the naked eye. Furthermore, using a moist test strip to wipe a trace amount of nitrite solid (5 ng to 1 mg), a clear green emission can also be observed generally within 10 s (Figure 3d). It should be noted that nitrite solid with a mass of 5 ng is enough to switch on the obvious green emission. This superior detection performance can be attributed to the limited diffusion of the solid aggregates inducing an intense signal in a restricted area.

To avoid the interference of common solvents on the detection performance of the test strip, >20 types of interferents, including strong redox active, coloured, and nitro compounds, in the form of a solution and solid were selected for comparison (Figure 3e,f). Upon a quick observation, except for Na_2_CO_3_ and CuCl_2_, all the substances show no obvious effect on the green emission induced by nitrite. Na_2_CO_3_ consumes the acid and leads to the recovery of the blue emission. However, this emission does not affect the green emission. While for CuCl_2_, its green colour does not interfere with the collection of the characteristic signal. Furthermore, the test strips also could realize precise and rapid qualitative detection of nitrite in foods (Figure S29, Supporting Information). This result further proved that the *ortho*‐BT‐based test strip is highly reliable for the practical detection of nitrite, confirming the *ortho*‐binding induced specific intramolecular coupling with nitrite.

### 2.4 *Ortho*‐BT‐Embedded Hydrogel‐Based Sensor for Nitrite Microparticulates Detection

**Figure 4:**
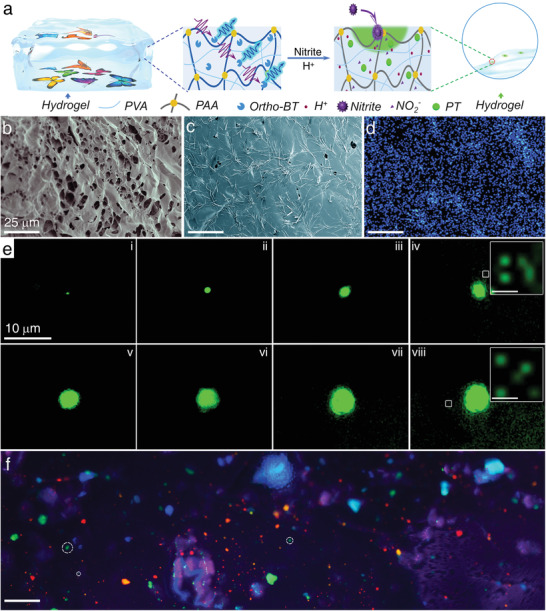
a) Schematic of the structure and transmittance of the hydrogel device and the fluorescence detection of nitrite airborne microparticulates. Microstructure of the hydrogel b) without and c) with *ortho*‐BT, and d) corresponding EDS mappings of sulfur. e) Dark‐field fluorescence images of the hydrogel device upon the addition of nitrite particulates with various sizes (Insets: enlarged fluorescence images; scale bar = 0.5 µm). f) Dark‐field fluorescence images of the hydrogel device upon adhering various fluorescent or nonfluorescent substances (Scale bar = 50 µm; some of the green emission dots switched on by nitrite microparticulates are marked with white dash line circles). (Excitation source: 365 nm LED; Objective magnification: 20–60 ×).

Either the industrial use of nitrite or making IEDs with nitrite as the raw material, microparticulates can be separated from solids and suspended in air.^[^ ^18^ ^]^ Thus, the detection of nitrite airborne microparticulates may be an effective way for industrial safety monitoring and explosive detection. Therefore, how to seize these microparticulates and make the ultraweak optical signal detected is of great importance.^[^ ^19^ ^]^ However, the size of the fibers in filter paper being opaque in the range of several to tens of microns will block the fluorescence signal. Thus, embedding *ortho*‐BT in a platform with a nanoscale transparent skeleton facilitates the transmission and recording of the fluorescence signal without any loss in its intensity. Thus, a polyacrylamide and poly(vinyl alcohol) (PAA/PVA) hydrogel with an interpenetrated network and high transmittance was used as a substrate to embed *ortho*‐BT and enhance the detection performance toward nitrite microparticulates (**Figure** **4**a; Figure S30, Supporting Information). The honeycombed pore structure was evenly spread over the framework and facilitated the uniform distribution of *ortho*‐BT (Figure 4b–d), which was highly desirable for the detection of microparticulates.

This hydrogel device was applied toward the detection of nitrite microparticulates suspended in the air and a series of fluorescence images focusing on particles with different sizes were captured (Figure 4e). It is considered that the real size of the nitrite microparticulate will be smaller than the size of the fluorescent dot observed due to the dissolution and dispersion of nitrite itself or the reaction products. Thus, the real size of the nitrite microparticulates were estimated to be in the range of a few hundreds of nanometres to several micrometers. From the fluorescence images, it was observed that the area of the green emission obviously increases with the size of the nitrite microparticulates. It was estimated that when the size of the fluorescent dot was ≈0.97 µm, the mass of the nitrite microparticulate could be as low as 1 pg based on the spherical approximation (Figure 4e(i)). Further analysis of these images revealed that the tiny fluorescent dots accompanied with the microsized emission can also be distinguished from the background (typically enlarged as the insets of Figure 4b(iv),(viii)). Generally, the number of these tiny spots increases with the enlargement of the main emission dot. The reason for the occurrence of these tiny spots can be attributed to the electrostatic interactions formed between the microsized master nitrite particles and nanosized subsidiary nitrite particles. It is noteworthy that the size of these tiny fluorescent spots was ≈200 nm, which is close to the resolution limit of fluorescence microscopy. Thus, a detection performance for a mass of ≈2.2 fg was realized by the hydrogel device. To the best of our knowledge, this is much more sensitive than other previously reported nitrite sensors. Undoubtedly, the ultrasensitive detection performance toward nitrite microparticulates can be attributed to not only the *ortho*‐binding induced fluorescent turn‐on mode of action, but also the hydrogel substrate boosting the signal intensity.

In addition, while considering actual complex application scenarios, that is, various colourful or fluorescent substances will simultaneously exist in the air, it is inevitable that these interferents will also be gathered on the surface of the hydrogel device. To precisely simulate this situation, the hydrogel device was used to capture a microparticulate mixture consisting of >7 fluorescent substances with different emissions and nitrite. An amazing rosy cloud‐like fluorescent image with numerous red, blue, purple, orange, yellow, and green emission dots or cloud clusters were observed (Figure 4f). Some of the green fluorescent spots were marked by a white frame. It is noteworthy that although in this extremely complex situation, green fluorescent dots of ≈2 µm were observed with no interference. This result further demonstrates the advantage of the hydrogel device in efficiently enhancing the detection sensitivity toward nitrite even under extremely complex circumstances.

### 2.5 Portable Detection Platform of Nitrite Employing Paper Test Strip, Encapsulated Paper, and Hydrogel Device

**Figure 5:**
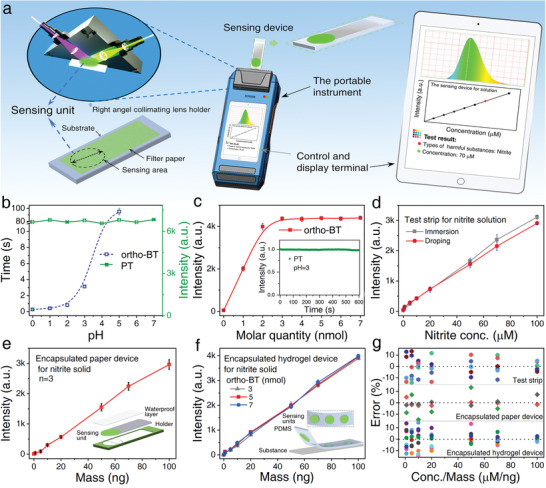
a) Schematic of the working principle of a portable fluorescence detection platform. b) Response time (blue) of *ortho*‐BT toward nitrite and emission intensity (green) of **PT** as a function of pH. c) The change in the fluorescence intensity of ortho‐BT toward nitrite at a concentration of 2 × 10^−9^nmol (Inset: fluorescence stability of **PT** produced by *ortho*‐BT (5 × 10^−9^nmol) at pH 3). d) Fluorescence intensity of the test strips as a function of the nitrite concentration (0–100 × 10^−6^ m). e) Fluorescence intensity of the encapsulated paper device as a function of the nitrite mass (0–100 ng); the device structure is shown in the inset. f) Fluorescence intensity of the encapsulated hydrogel devices containing ortho‐BT (3, 5, and 7 × 10^−9^nmol) as a function of the nitrite mass (0–100 ng); the device structure is shown in the inset. g) Statistical errors for the three devices shown in (d)–(f). All the spectral data of Figure 5 were obtained through the portable fluorescence sensing platform. (*λ*_ex_= 365 nm; *λ*_em_= 530 nm; Slits: 2 nm; Integral time: 100 ms; Spectrometer: Maya 2000pro).

To further prove the applicability of the proposed fluorescent probe design strategy for the rapid, on‐site, and semiquantitative measurement of nitrite, a portable detection platform consisting of a grating spectrometer, detection module and excitation source, and controller was constructed (**Figure** **5**a). The qualitative and semiquantitative results can be obtained upon acquisition and processing of the spectrum. In addition, the detection performance of this designed *ortho*‐BT could be further verified and evaluated based of this data.

The paper test strip, encapsulated paper device, and encapsulated hydrogel device can respond to nitrite in <3 s when the pH was <3 (Figure 5b, blue line; Figures S31 and S32, Supporting Information) and considering the operating requirement, the pH value of all the devices was adjusted to 3. In addition, the readout was further confirmed to be unaffected by the change in the pH of the sensing device in the range of 0–7 (Figure 5b, green line). The signal also shows long term stability, indicating the great stability of the sensing device and the portable detection platform. Furthermore, by considering the practical need for detecting a nitrite quantity as high as 2 nmol (NaNO_2_, 137.9 ng), the sensing device should embed 2 nmol of *ortho*‐BT to fully react and thus, give the absolute quantity of nitrite (Figure 5c; Figure S32c, Supporting Information). It should be noted that the fluorescence intensity of the device is very stable and will not change once the quantity of *ortho*‐BT exceeds 2 nmol (Figure 5c inset). Thus, to further consider of the actual detection range, the amount of *ortho*‐BT in the individual sensing device was chosen to be 5 nmol in the following experiment.

Under the optimized conditions, the spectra for three types of detection devices, including the paper test strip, encapsulated paper device, and encapsulated hydrogel device, toward a nitrite solution or solid were automatically recorded and processed using this portable detection platform. When dropping a nitrite solution (1–100 × 10^−6^ m) on the paper test strip or immersing it into the nitrite solution, the fluorescence intensity of the paper test strip increases linearly upon increasing the nitrite concentration (Figure 5d; Figure S33a,b, Supporting Information). It should be noted that the sampling method itself does not obviously influence the final judgement of the nitrite concentration since the two curves overlap well with each other when the nitrite concentration is <50 × 10^−6^ m and are only slightly separated when the nitrite concentration is >50 × 10^−6^ m. Furthermore, the encapsulated paper device was used to detect nitrite solid within the range of 1–100 ng by wiping (Figure 5e; Figure S33c, Supporting Information). An almost linear relationship between the fluorescence intensity and the mass can also be observed. Besides, due to the maximum fluorescence error being within 11.8%, it can be concluded that the judgement of the nitrite solid mass is basically consistent despite the possible mass difference in the sampling batches. The fluorescence intensity of the encapsulated hydrogel device shows an even more perfect linear relationship with the mass of nitrite solid (Figure 5f; Figure S34, Supporting Information). Moreover, embedding different amounts of *ortho*‐BT (3, 5, and 7 nmol) did not obviously influence the final judgement of the nitrite mass since the three curves almost overlap each other. The largest error is located at the standard intensity given by the encapsulated hydrogel device containing 5 nmol of the *ortho*‐BT probe toward 100 ng of nitrite solid; the other two devices containing 3 and 7 nmol of the *ortho*‐BT probe show a nitrite solid mass of 96 and 104 ng, respectively.

To further verify the reliability of the three devices together with the portable detection platform, the measurement errors obtained for all the devices in the detection range studied were statistically analysed (Figure 5g). Despite the device form, the measurement error for the different sampling methods, sampling batches, and amount of embedded probe molecules could be restrained within 11.8%, 13.5%, and 14.8%, respectively. The above results clearly indicate that the designed *ortho*‐BT is highly stable and reliable for the practical application of the portable detection platform and thus, will facilitate the rapid, on‐site, and semiquantitative detection of nitrite.

## 3 Conclusion

In summary, we have demonstrated the precise modulation of the fluorophore binding sites is of great importance toward regulating the sensing properties of the probe and to further achieve an excellent identification capability toward the target analyte. Stable and reliable *ortho*‐BT prepared via the introduction of benzothiazole to the *ortho*‐position of aniline not only exhibits high reactivity toward trace amounts of nitrite and form a specific cyclization reaction product, but also achieved a fluorescent turn‐on mode of action to maximize the detection efficiency, including a visual sensitive green emission, low detection limit, rapid detection time, and excellent specificity. The proposed probe design strategy has been proven to be reliable for the exploration of a portable detection platform, which is safe and simple for the rapid, on‐site, and semiquantitative detection of trace amounts of nitrite. We believe that the present strategy will be a promising candidate for the realization of the in‐field detection of trace amounts of hazardous substances.

## 4 Experimental Section

All materials, instrumentation, and methods are provided in the Supporting Information.

## Conflict of Interest

The authors declare no conflict of interest.

## Supporting information

Supporting Information
