# Supplementary material for: Ultrasensitive, Specific, and Rapid Fluorescence Turn‐On Nitrite Sensor Enabled by Precisely Modulated Fluorophore Binding
Source: Adv Sci (Weinh). 2020 Nov 13;7(24):2002991. doi: 10.1002/advs.202002991 (PMC7740093; doi:10.1002/advs.202002991)
Supplement: Supplementary file 1 — Supporting Information [file ADVS-7-2002991-s001.pdf]

© 2020 Wiley-VCH GmbH

ADVANCED SCIENCE
Open Access

Supporting Information

for *Adv. Sci.*, DOI: 10.1002/advs.202002991

Ultrasensitive, Specific and Rapid Fluorescence Turn-on Nitrite Sensor Enabled by Precisely Modulated Fluorophore Binding

*Zhiwei Ma, Jiguang Li, Xiaoyun Hu, Zhenzhen Cai, and Xincun Dou**

© 2020 Wiley-VCH GmbH

Supporting Information

Title

Ultrasensitive, Specific and Rapid Fluorescence Turn-on Nitrite Sensor Enabled by Precisely Modulated Fluorophore Binding

*Zhiwei Ma, Jiguang Li, Xiaoyun Hu, Zhenzhen Cai, and Xincun Dou**

Experimental Section

Materials and characterization

Unless otherwise noted, all reagents and materials were obtained from commercial sources and all were used without further purification. Acrylamide (AAm) and N,N′-methylenebisacrylamide (MBA) were purchased from Sigma-Aldrich. Polydimethylsiloxane Sylgard 184 (PDMS) was purchased from Dow Corning. Triphenyl phosphite (TPP), 2-Aminothiophenol, Tetrabutylammonium bromide (TBAB), Cetyltrimethylammonium Bromide (CTAB), 2-aminoterephthalic acid, 2-amino-5-methylbenzoic acid, 2-amino-5-chlorobenzoic acid, 3-aminobenzoic acid, 4-aminobenzoic acid, poly (vinyl alcohol) (PVA) (MW 60,000) and glutaraldehyde solution (GD) were purchased from Aladdin Chemical Reagent Ltd., and all the organic solvents and inorganic salts were purchased from Sinopharm Chemical Reagent Ltd., includes potassium persulfate (KPS) and hydrochloric acid (HCl), etc. Qualitative filter paper was purchased from Titan. Solvents for synthesis and optical studies were used in analytical grade and HPLC grade, respectively.

Using tetramethylsilane (TMS) as internal standard, ^{1}H NMR and ^{13}C NMR spectra were measured on Varian high resolution 400 MHz superconducting NMR Spectrometer. Mass spectra of fluorescent probes and products were determined by liquid chromatography-ion trap mass spectrometry (Agilent GC-MS 7890A/5977B). Ultraviolet-visible absorption spectra were measured on Hitachi UV-3900 ultraviolet-visible spectrophotometer and fluorescence spectra were measured

on Edinburgh FLS1000 fluorescence spectrophotometer. Laser confocal Raman, micro-region fluorescence-reflection-transmission spectroscopy and imaging system was constructed from inverted fluorescence microscope, laser and Andor fluorescence spectrometer. The morphology and structure of filter paper and hydrogel were characterized using a Field-emission Scanning Electron Microscope (FE-SEM JEOL JSM-7610F Plus, Japan) operating at 4.0–6.0 kV. Elemental analysis was performed by an Energy-Dispersive Spectroscopy System (OXFORD X-Max 50) equipped on the FE-SEM. Dark-field images were carried out on Nikon Ti-E inverted florescence microscope to detect nitrite particles, and the optical images were obtained by the Nikon camera (d610). Grating spectrometer (Maya 2000 Pro) of ocean optics was used to build the portable optical detector.

1. Synthesis process of probes

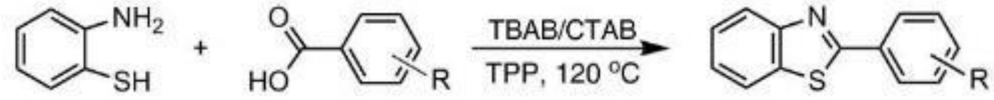

Scheme S1. Synthetic procedures of probes

1. 2-(2-amino-4-carboxyphenyl) benzothiazole (ortho-BT)

A mixture of 2-aminoterephthalic acid (726 mg, 4.0 mmol), 2-aminothiophenol (500 mg, 4.0 mmol), CTAB (3.49 g, 9.6 mmol) and TPP (3.12 g, 9.6 mmol) were mixed well in the 50 mL double-mouth round-bottom flask, and the mixture solution was stirred overnight at 120 °C. Precipitation of the products were carried out from the viscous solution by adding a mixture of cold acetone. The resulting solid products were filtered off, and then the crude products were purified by column chromatography to give the desired probe 2-(2-amino-4-carboxyphenyl) benzothiazole (ortho-BT) with 48% yield. ^{1}H NMR (400 MHz, DMSO-d_6) δ 8.40 (s, 1H), 8.07 (d, J = 7.8 Hz, 2H), 8.00 (d, J = 8.0 Hz, 2H), 7.57 (d, J = 8.1 Hz, 2H), 7.50 (dd, J = 9.1, 6.1 Hz, 3H), 7.41 (t, J = 7.6 Hz, 2H), 7.30 (s, 3H), 7.20 (d, J = 8.4 Hz, 2H). ^{13}C NMR (101 MHz, DMSO-d_6) δ 168.31, 167.45, 153.53, 147.84, 133.58, 133.09, 130.56, 127.04, 125.98, 122.86, 122.32, 118.13, 116.34. EI-MS: $[M]^+$ Calcd. for 270.3; Found 270.0.

2. 2-(2-amino-5-methylphenyl) benzothiazole

A mixture of 2-amino-5-methylbenzoic acid (604 mg, 4.0 mmol), 2-aminothiophenol (500 mg, 4.0 mmol), TBAB (1.163 g, 4.8 mmol) and TPP (1.06 g, 4.8 mmol) were mixed well in the 25 mL round-bottom flask, and the mixture solution was stirred overnight at 120 °C. Precipitation of the crude products were carried out from the viscous solution by adding cold methanol. The resulting solid products were filtered off and washed with cold deionized water, then the crude products were purified by column chromatography to give the desired probe 2-(2-amino-5-methylphenyl) benzothiazole with 83% yield. ^{1}H NMR (400 MHz, DMSO-d_6) δ 8.00 (d, J = 7.9 Hz, 1H), 7.94 (d, J = 8.1 Hz, 1H), 7.48 – 7.40 (m, 1H), 7.34 (dd, J = 11.6, 4.3 Hz, 2H), 7.09 (s, 2H), 7.03 – 6.98 (m, 1H), 6.98 (s, 1H), 6.77 (s, 1H), 2.17 (s, 3H); ^{13}C NMR (101 MHz, DMSO-d_6) δ 169.21, 153.69, 146.00, 133.39, 132.85, 129.83, 126.78, 125.41, 124.49, 122.40, 122.09, 117.23, 113.43, 20.35. EI-MS: EI-MS: $[M]^+$ Calcd. for 240.0; Found 240.1.

3. 2-(2-amino-5-chlorophenyl) benzothiazole

A mixture of 2-amino-5-chlorobenzoic acid (684 mg, 4.0 mmol), 2-aminothiophenol (500 mg, 4.0 mmol), TBAB (1.163 g, 4.8 mmol) and TPP (1.06 g, 4.8 mmol) were mixed well in the 25 mL round-bottom flask, and the mixture solution was stirred overnight at 120 °C. Precipitation of the crude products were carried out from the viscous solution1 by adding cold methanol. The resulting solid products were filtered off and washed with cold deionized water, then the crude products were purified by column chromatography to give the desired probe 2-(2-amino-5-chlorophenyl) benzothiazole with 83% yield. ^{1}H NMR (400 MHz, DMSO-d_6) δ 8.08 (d, J = 7.9 Hz, 1H), 8.01 (d, J = 8.0 Hz, 1H), 7.56 (d, J = 2.3 Hz, 1H), 7.50 (t, J = 7.7 Hz, 1H), 7.47 – 7.33 (m, 3H), 7.22 (dd, J = 8.8, 2.4 Hz, 1H), 6.90 (d, J = 8.9 Hz, 1H). ^{13}C NMR (101 MHz, DMSO-d_6) δ 167.64, 153.47, 146.95, 132.93, 131.89, 128.72, 127.02, 125.89, 122.73, 122.28, 118.90, 118.85, 114.51. EI-MS: $[M]^+$ Calcd. for 260.0; Found 260.7.

4. 2-(3-aminophenyl) benzothiazole (meso-BT)

A mixture of 3-aminobenzoic acid (548 mg, 4.0 mmol), 2-aminothiophenol (500 mg, 4.0 mmol), TBAB (1.163 g, 4.8 mmol) and TPP (1.06 g, 4.8 mmol) were mixed well in the 25 mL round-bottom

flask, and the mixture solution was stirred overnight at 120 °C. Precipitation of the crude products were carried out from the viscous solution by adding cold methanol. The resulting solid products were filtered off and washed with cold deionized water, then the crude products were purified by column chromatography to give the desired probe 2-(3-aminophenyl) benzothiazole (meso-BT) with 45% yield. ^{1}H NMR (400 MHz, DMSO-d_6) δ 8.08 (d, J = 8.0 Hz, 1H), 7.98 (d, J = 8.0 Hz, 1H), 7.49 (dd, J = 11.2, 4.1 Hz, 1H), 7.43 – 7.38 (m, 1H), 7.32 (s, 1H), 7.18 – 7.09 (m, 3H), 6.71 (d, J = 6.6 Hz, 1H), 5.87 (s, 1H). ^{13}C NMR (101 MHz, DMSO-d_6) δ 168.64 (s), 153.98 (s), 149.87 (s), 134.69 (s), 133.83 (s), 131.94 (s), 130.23 (s), 126.92 (s), 125.71 (s), 123.09 (s), 122.66 (s), 117.26 (s), 115.09 (s), 112.28 (s). EI-MS: $[M]^+$ Calcd. for 226.0; Found 226.2.

5. 2-(4-aminophenyl) benzothiazole (para-BT)

A mixture of 4-aminobenzoic acid (548 mg, 4.0 mmol), 2-aminothiophenol (500 mg, 4.0 mmol), TBAB (1.163 g, 4.8 mmol) and TPP (1.06 g, 4.8 mmol) were mixed well in the 25 mL round-bottom flask, and the mixture solution was stirred overnight at 120 °C. Precipitation of the crude products were carried out from the viscous solution by adding cold methanol. The resulting solid products were filtered off and washed with cold deionized water, then the crude products were purified by column chromatography to give the desired probe 2-(4-aminophenyl) benzothiazole (para-BT) with 91% yield. ^{1}H NMR (400 MHz, DMSO-d_6) δ 7.98 (d, J = 7.9 Hz, 1H), 7.85 (d, J = 8.1 Hz, 1H), 7.72 (d, J = 8.5 Hz, 2H), 7.41 (t, J = 7.7 Hz, 1H), 7.30 (t, J = 7.3 Hz, 1H), 6.63 (d, J = 8.5 Hz, 2H), 5.85 (s, 2H). ^{13}C NMR (101 MHz, DMSO-d_6) δ 168.54, 154.30, 152.60, 134.09, 129.81, 129.19, 126.64, 124.73, 122.32, 122.15, 120.44, 113.97. EI-MS: $[M]^+$ Calcd. for 226.0; Found 226.1.

6. Compound PT

The hydrochloric acid solution was added by dropping into a solution of ortho-BT (50 mg, 0.185 mmol) with acetone (20 mL) as the solvent until the fluorescence was quenched, then sodium nitrite (35 mg, 0.51 mmol) was added. This mixture was stirred for 1 min at room temperature. After the reaction finished, the solvents were removed under reduced pressure and the resulting solid was purified by column chromatography to give the pure product (6H-benzo[e]benzo[4,5]thiazolo[3,2-

c][1,2,3]triazine-3-carboxylic acid, **PT**). ^{1}H NMR (400 MHz, DMSO-d_6) δ 12.95 (s, 1H), 8.10 (d, J = 8.0 Hz, 1H), 8.03 (d, J = 8.1 Hz, 1H), 7.72 (d, J = 8.2 Hz, 1H), 7.51 (dd, J = 8.3, 5.0 Hz, 3H), 7.42 (t, J = 7.1 Hz, 1H), 7.14 (dd, J = 8.2, 1.6 Hz, 1H). ^{13}C NMR (101 MHz, DMSO-d_6) δ 157.12 (s), 154.41 (s), 153.70 (s), 153.51 – 153.31 (m), 151.93 (s), 148.85 (s), 137.39 (s), 136.85 (s), 134.94 (s), 129.75 (s), 127.06 (s), 124.54 (s). EI-MS: $[M]^+$ Calcd. for 283.0; Found 283.3.

7. PAA/PVA double network hydrogel

For a typical synthesis of PAA/PVA double network hydrogel, the hydrogel contains two interpenetrating networks: PAA and PVA. The PAA precursor was synthesized from a 4 M AAm aqueous solution mixture with 4 mM MBA and 4 mM KPS. And the PVA precursor was synthesized from a 1 mM PVA and 0.016 M GD mixture aqueous solution. These two precursors were mixed with a volume ratio of 3:1 and then HCl was added in the mixture solution till a pH value of 2 was reached, and then stirred for 1 hour in ambient air to form a hydrogel prepolymer solution. The solution was then heated to 60 °C and maintained for 1 hour, then washed and stored in deionized water for device fabrication.

8. Preparation of PDMS substrate

PDMS substrates were prepared by pouring a mixture of Sylgard 184 base and curing agents (10:1 by weight) into a prepared resin mold, and treated at 80 °C for 3 h. The resulting PDMS substrates surface with a predetermined pattern were washed several times, then dried in air and stored in sealed bag.

2. Preparation of samples and testing process

(1) **Preparation of hydrochloric acid stock solution (pH = 2.88):** The concentrated hydrochloric acid and acetone were mixed by the volume ratio of 1:1, and then the mixed solution was diluted by the mixture of deionized water and acetone with a volume ratio of 1:1 until the pH value reached to 2.88.

(2) **Preparation of ortho-BT probe solution (2 mM):** 270 mg ortho-BT was dissolved in 0.5 L of

hydrochloric acid stock solution to get the ortho-BT probe solution (2 mM). The pH value is still 2.88.

(3) Preparation and testing of nitrite solution

Nitrite stock solution: 10 mmol sodium nitrite was dissolved in deionized water to prepare the nitrite stock solution with a concentration of 10 mM. Then the solution was diluted with deionized water to obtain various concentrations of nitrite solutions.

Spectral response testing: 1 mL of nitrite solution (4, 20, 40, 80, 160, 240, 320, 400, 800, 1600, 2400, 3200, and 4000 μM, respectively) was added into 3 mL of the ortho-BT probe solution in a cuvette. The final pH value could be well maintained to be around 3. The time-dependent spectral characteristics were measured and recorded with the portable fluorescence sensing platform. Fluorescence detection performance of nitrite was recorded with Edinburgh FLS1000 fluorescence spectrophotometer.

(4) Preparation and testing of trace analytes

Analyte stock solutions (4 and 8 mM): NH_4NO_3, Urea, Na_2SO_3, Na_2S, $CuCl_2$, Na_2SO_4, KI, $FeSO_4$, $KMnO_4$, H_2O_2, $FeCl_3$, $NaClO_4$, NaClO, Na_2CO_3, CH_4N_2S, KCl, Na_3PO_4, $NaNO_3$, $AgNO_3$, $AlCl_3$, $BaCl_2$, $Bi(NO_3)_3$, $CaCl_2$, $Cd(CH_3CO_2)_2$, $CoCl_2$, Cs_2CO_3, $Ga(NO_3)_3$, HgCl, $In(NO_3)_3$, $K_2Cr_2O_7$, $La(NO_3)_3$, $MgCl_2$, $Pb(CH_3CO_2)_2$, $SnCl_2$, $SnCl_4$, $Ti_2(SO_4)_3$, WCl_6, and $Zn(CH_3CO_2)_2$ were dissolved in deionized water to prepare the analyte stock solution with a concentration of 4 and 8 mM. Trinitrotoluene (TNT), picric acid (PA), and nitromethane (NT) solutions (4 and 8 mM) with acetone as the solvent were prepared respectively.

Analyte powders: Solid particles of various analytes ($NaNO_2$, NH_4NO_3, Urea, Na_2SO_3, Na_2S, $CuCl_2$, Na_2SO_4, KI, $FeSO_4$, $KMnO_4$, $FeCl_3$, $NaClO_4$, NaClO, $NaIO_4$, Na_2CO_3, CH_4N_2S, KCl, Na_3PO_4, TNT, PA, and NT) were ground into fine powder using an agate mortar for the microparticulate detection and anti-interference detection performance test.

Cautions! Energetic materials should be handled very carefully in small quantities be ground gently!

Trace sodium nitrite powders: Different concentrations of sodium nitrite solution were firstly prepared to be 0.25, 0.5, 1, 2.5, 5, 25, 50, 500, 1500, 2500, and 5000 mg/L, respectively. Then, 20 μL of the above sodium nitrite solution was measured with a pipette gun and added on a ceramic plate. After natural air drying, the solid sodium nitrite with different masses from 5 ng-1 mg could be obtained.

Trace nitrite airborne microparticulates: Airborne microparticulates were created by finely grinding the mixed powders of 1 mg sodium nitrite solid and 1 g silica gel in an agate mortar and blowing them with air.

Selectivity testing process: The stock solution (4 mM, 1 mL) of certain analyte was added into a cuvette filled with 3 mL of the ortho-BT probe solution. After the reaction, the fluorescence spectra and images were measured and recorded. (λ_{ex} = 365 nm; Slits: 1 nm; λ_{em} = 530 nm; Slits: 0.3 nm; Spectrometer: Edinburgh FLS1000).

Anti-interference testing process: The mixture of the stock solution (8 mM, 0.5 mL) of certain analyte and nitrite stock solution (80 μM, 0.5 mL) was added into a cuvette filled with 3 mL of the ortho-BT probe solution. After the reaction, the fluorescence spectra and images were measured and recorded. (λ_{ex} = 365 nm; Slits: 1 nm; λ_{em} = 530 nm; Slits: 0.3 nm; Spectrometer: Edinburgh FLS1000).

3. Device fabrication and testing process

(1) Preparation and testing process of the test strip and encapsulated paper device

Preparation of test strip : Filter paper was immersed in the ortho-BT probe solution (2 mM) for 1 minute, dried in air, and stored at room temperature for next step use.

Test strips for nitrite solutions and solids detection in Figure 3: Different concentrations of sodium nitrite solution were firstly prepared to be 1, 5, 10, 20, 50, 100, 200, 400, 600, and 800 μM, respectively. Then, 20 μL of the above sodium nitrite solution was measured with a pipette gun and

added on a test strip. To detect trace nitrite solid, a test strip was used to wipe the prepared trace sodium nitrate solid powders. After that, the fluorescence images of the test strips were recorded with a digital camera.

Paper test strip for nitrite solutions and solids detection in Figure 5d: The paper test strip was dropped on with 20 μL sodium nitrite solutions (1, 5, 10, 20, 50, 70, and 100 μM) or immersed into these nitrite solutions, respectively. After that, the fluorescence spectra of the paper device were recorded with the portable detection platform.

Preparation of encapsulated paper device: The test strip was cut into 1 cm × 3 cm to completely fit with the groove on the prepared polypropylene substrate to ensure the consistency of all device structures. The whole device was covered with a waterproof layer to ensure the sensitive area be moist for wiping application.

Encapsulated paper device for nitrite solids detection in Figure 5e: An encapsulated paper device was used to wipe the prepared trace sodium nitrite solid powders (1, 5, 10, 20, 50, 70, and 100 ng). After that, the fluorescence spectra of the encapsulated device were recorded with the portable detection platform.

(2) Preparation of the hydrogel device and the encapsulated hydrogel device

Preparation of hydrogel device: The hydrogel was firstly immersed in the ortho-BT stock solution for 1 h, and then was brought out and stored in air to get the hydrogel device.

Hydrogel device for nitrite airborne microparticulates in Figure 4: A hydrogel device was used to collect the as prepared trace sodium nitrate solid powders. To dust the mixture powders with brush, then use the hydrogel device to collect the nitrite airborne microparticulates, and then, the fluorescence detection images of the hydrogel device were recorded with the dark field fluorescence microscope.

Preparation of encapsulated hydrogel device: The prepolymer solution was directly cured into the grooves on PDMS patterned substrate, followed by a heat treatment at 60 °C for 1 h. With a crosslinking process, the hydrogel was anchored onto the PDMS substrate, and then washed with deionized water. Ortho-BT stock solution was dropped onto the hydrogel area and encapsulated with a cap prepared by a commercial hard plastic film to obtain the encapsulated hydrogel device.

Encapsulated hydrogel device for nitrite solids detection in Figure 5f: An encapsulated hydrogel device was used to wipe the prepared trace sodium nitrite solid powders (1, 5, 10, 20, 50, 70, and 100 ng). After that, the fluorescence spectra of the encapsulated device were recorded with the portable detection platform.

Theoretical calculations

The geometries of singlet ground-state (S_0) were optimized by using density functional theory (DFT) with PBE1PBE[1]functional and a DEF2-TZVP[2] basis set. Gaussian 09 C.01 program package was employed for the calculation of the highest occupied molecular orbital (HOMO) and lowest unoccupied molecular orbital (LUMO) energies for optimized geometries of ortho-BT and **PT**. Molecular electrostatic potential surface was performed by using the Multiwfn program.[3] A colorized surface electrostatic potential distribution was shown by the VMD1.9.2 program.

Equipment research and development

The portable fluorescent detection instrument combined with highly sensitive sensors were used to achieve real time acquisition and recording of supersensitive spectral signals. The portable fluorescence detection platform is designed according to the actual device structure, which composes of controller (Tablet PC), highly sensitive grating spectrometer (Maya 2000 pro), detection module (P-C2 right-angle collimating lens holder) and LED excitation light source (365 nm UV lamp, 3 W). All the spectral data in Figure 5 and Figure 2d (fluorescence intensity) were obtained through this portable fluorescence sensing platform (λ_{ex} = 365 nm; λ_{em} = 530 nm; Slits: 2 nm; Integral time: 100 ms; Spectrometer: Maya 2000pro).

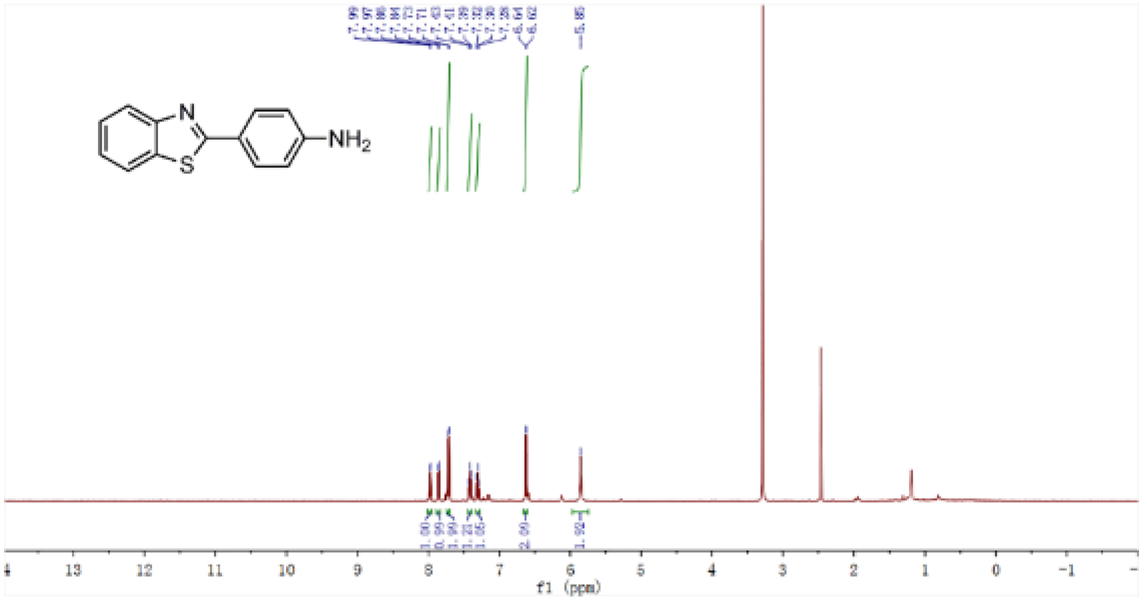

Figure S1. ^1H NMR spectra of para-BT (DMSO-d_6, 400 MHz).

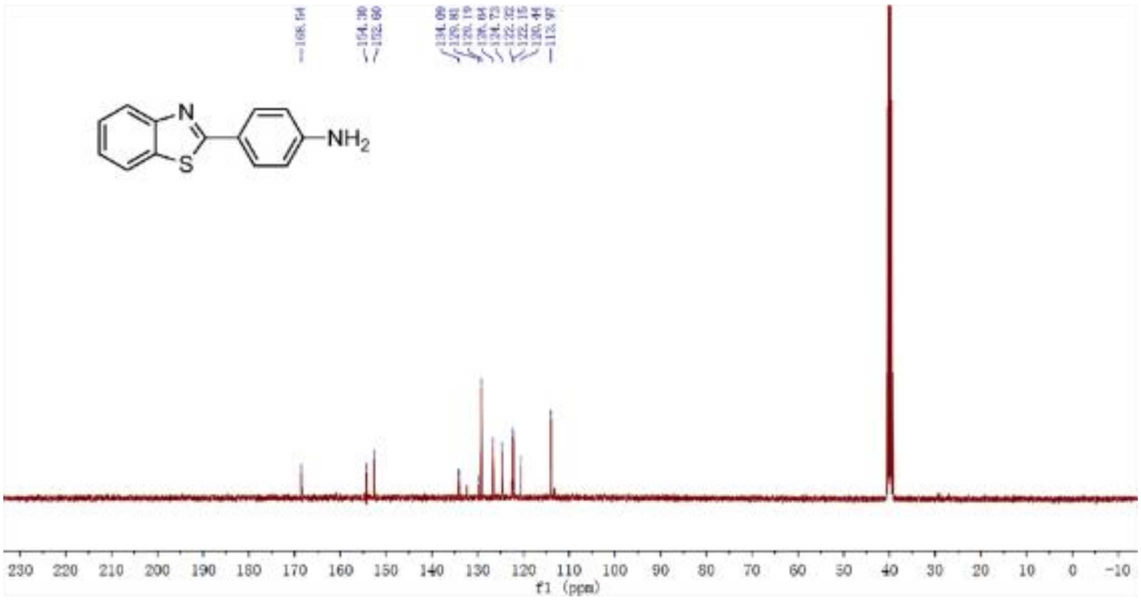

Figure S2. ^{13}C NMR spectra of para-BT (DMSO-d_6, 101 MHz).

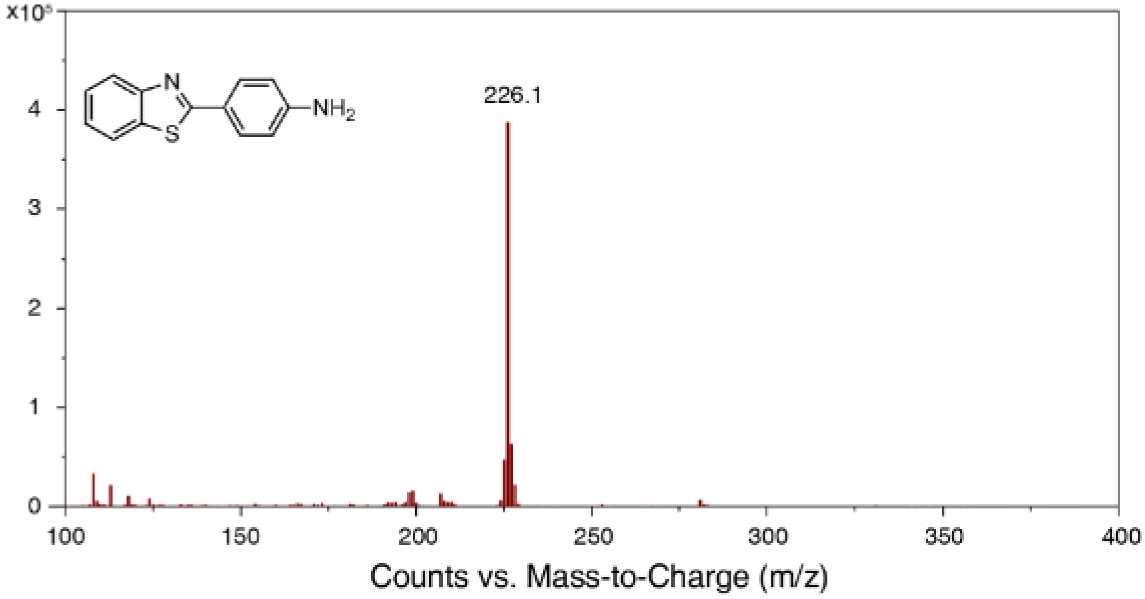

Figure S3. EI spectra of para-BT.

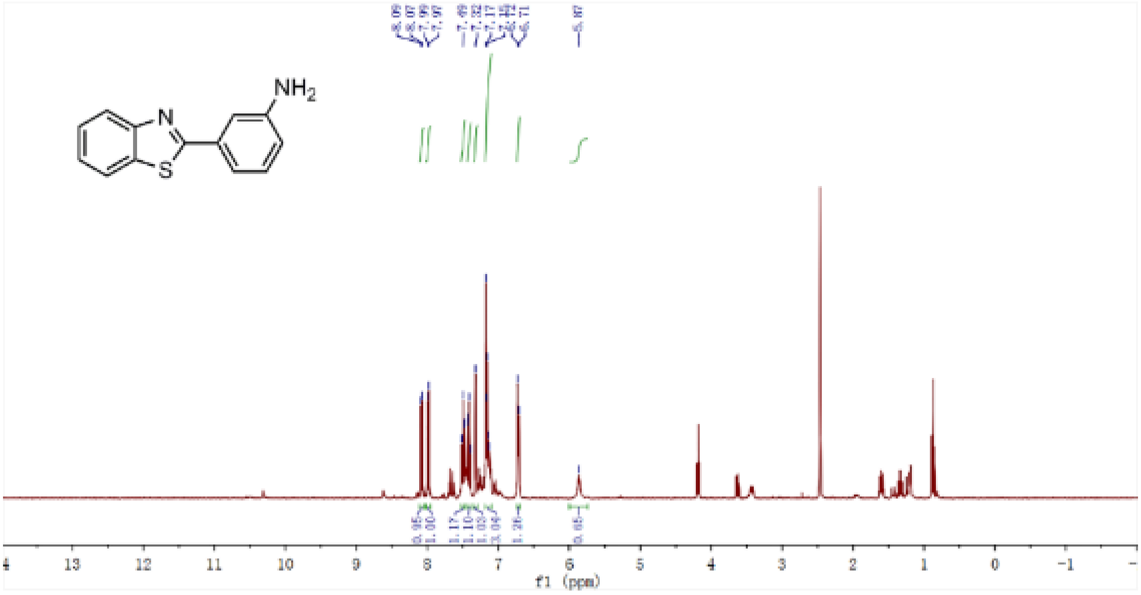

Figure S4. ^{1}H NMR spectra of meso-BT (DMSO-d_6, 400 MHz).

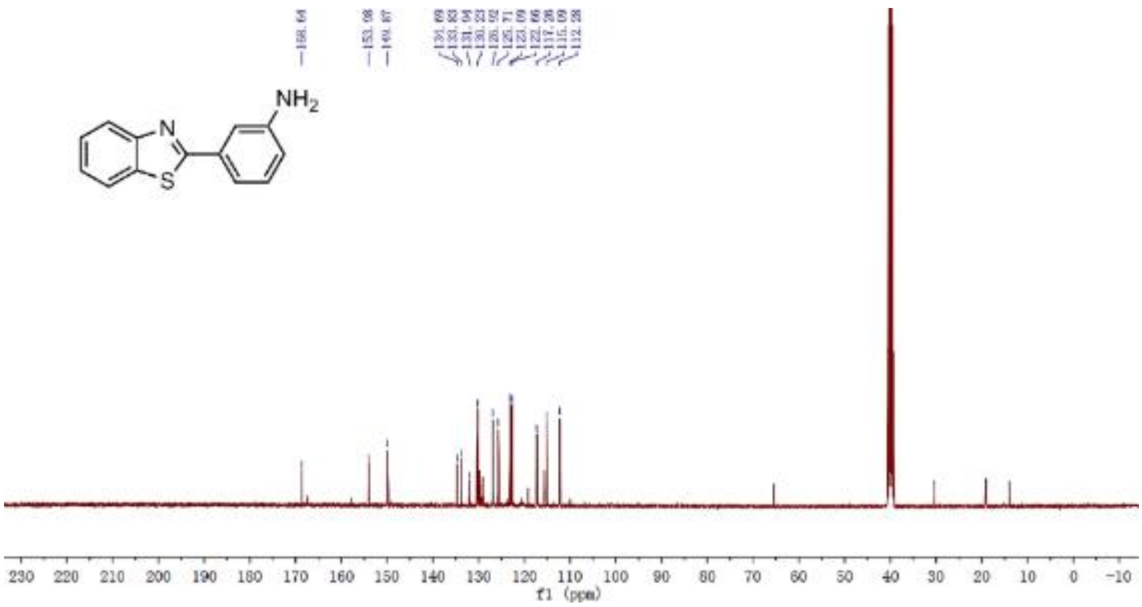

Figure S5. ^{13}C NMR spectra of meso-BT (DMSO-d_6, 101 MHz).

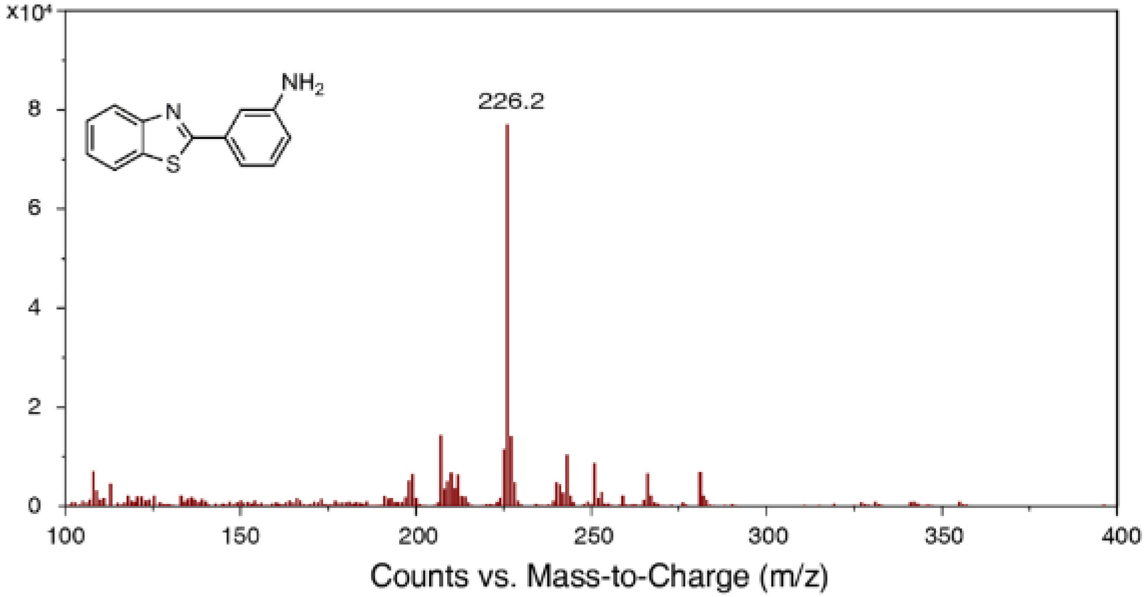

Figure S6. EI spectra of para-BT.

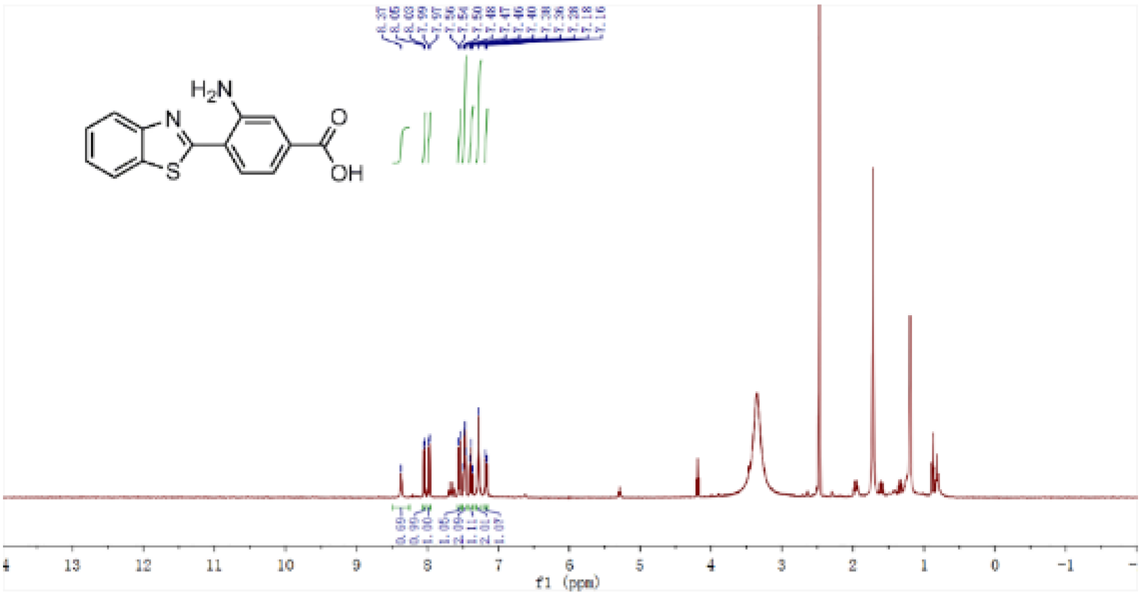

Figure S7. ^{1}H NMR spectra of ortho-BT (DMSO-d_6, 400 MHz).

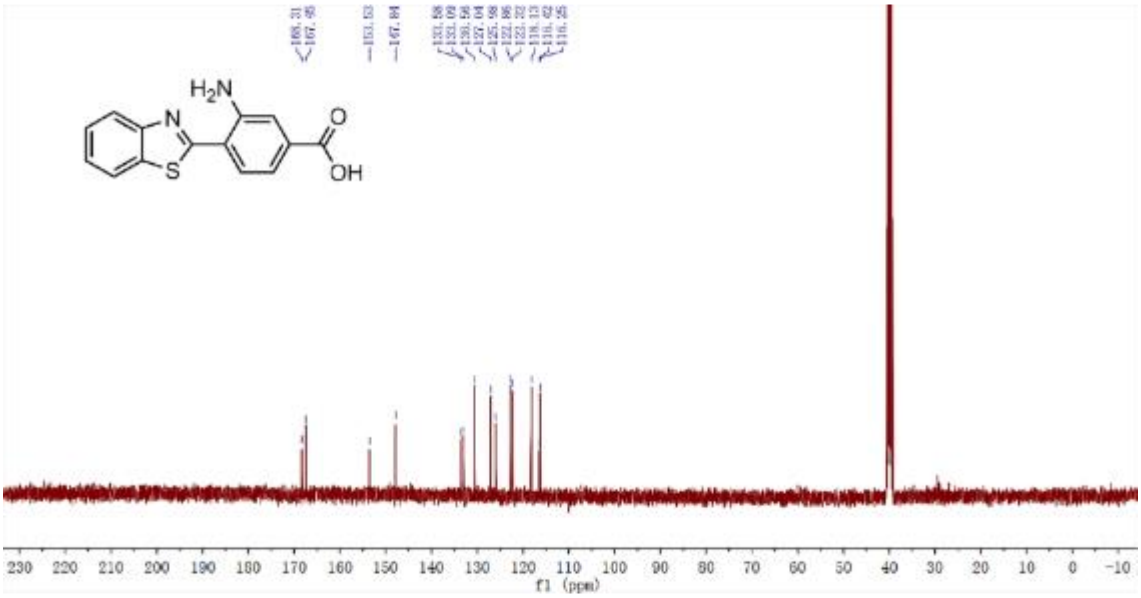

Figure S8. ^{13}C NMR spectra of ortho-BT (DMSO-d_6, 101 MHz).

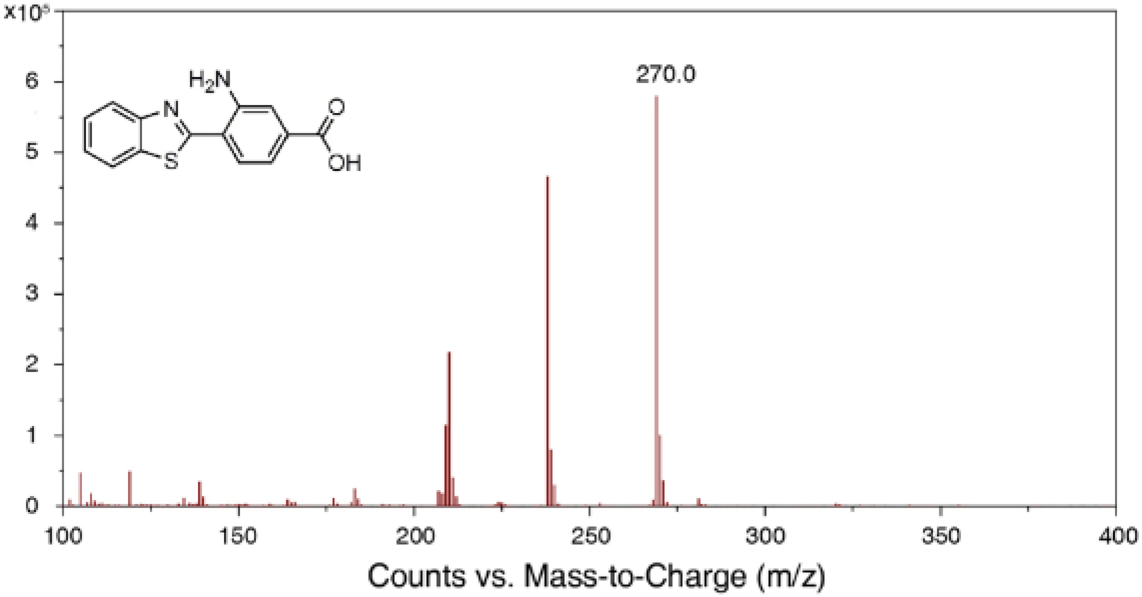

Figure S9. EI spectra of ortho-BT.

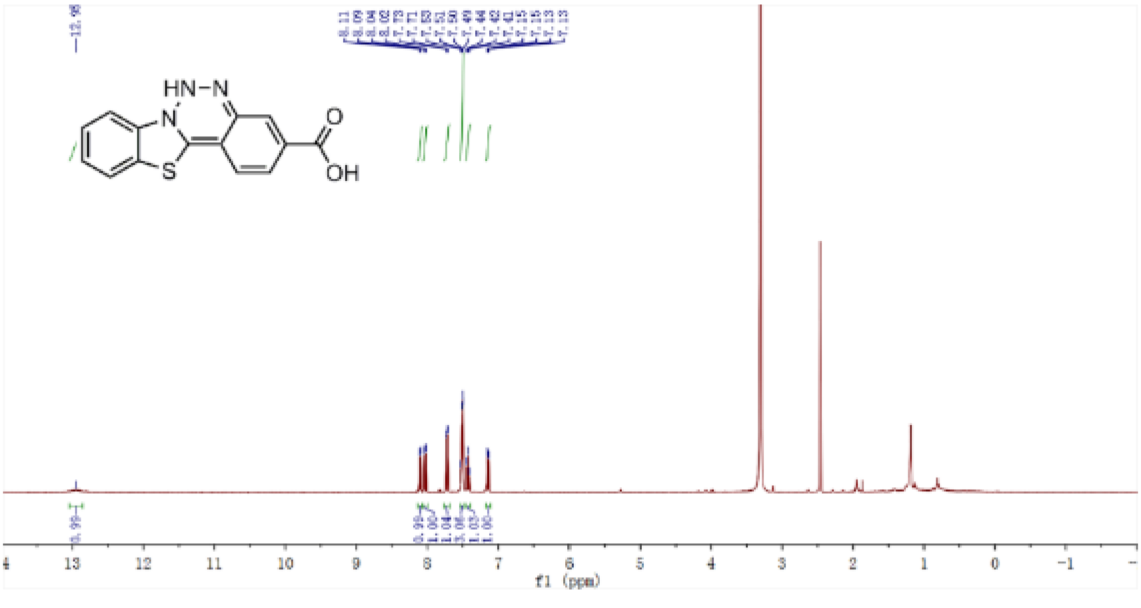

Figure S10. ^{1}H NMR spectra of **PT** (DMSO-d_6, 400 MHz).

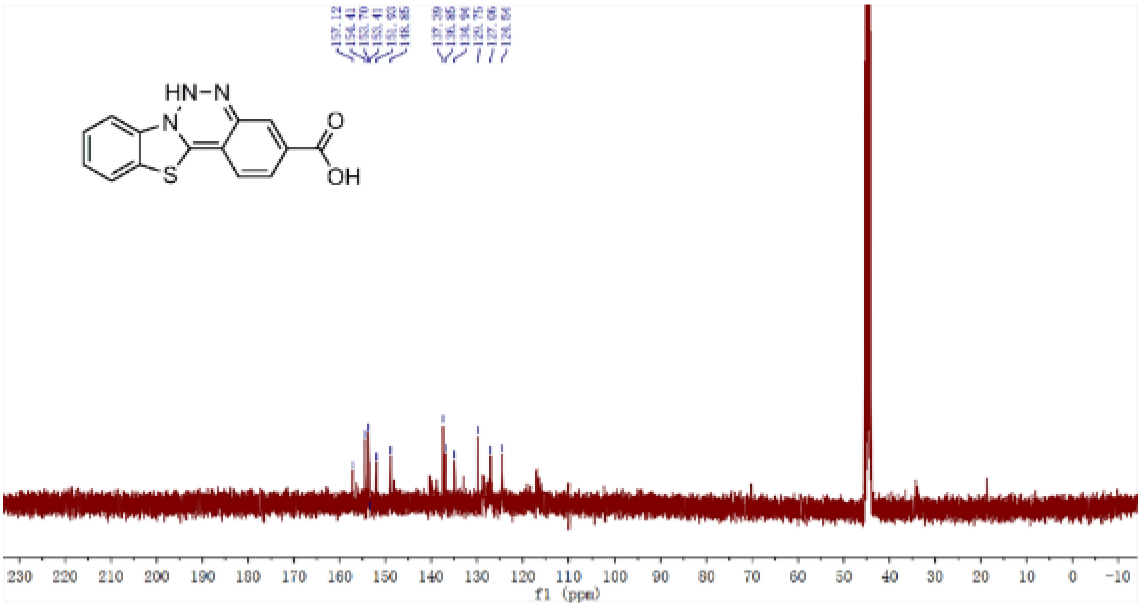

Figure S11. ^{13}C NMR spectra of **PT** (DMSO-d_6, 101 MHz).

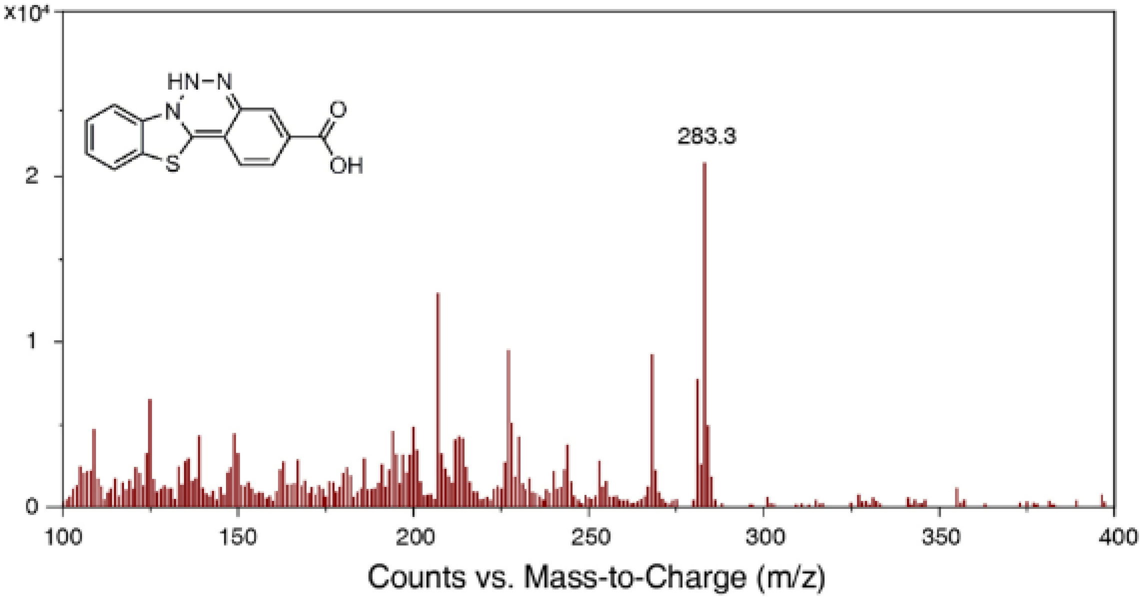

Figure S12. EI spectra of **PT**.

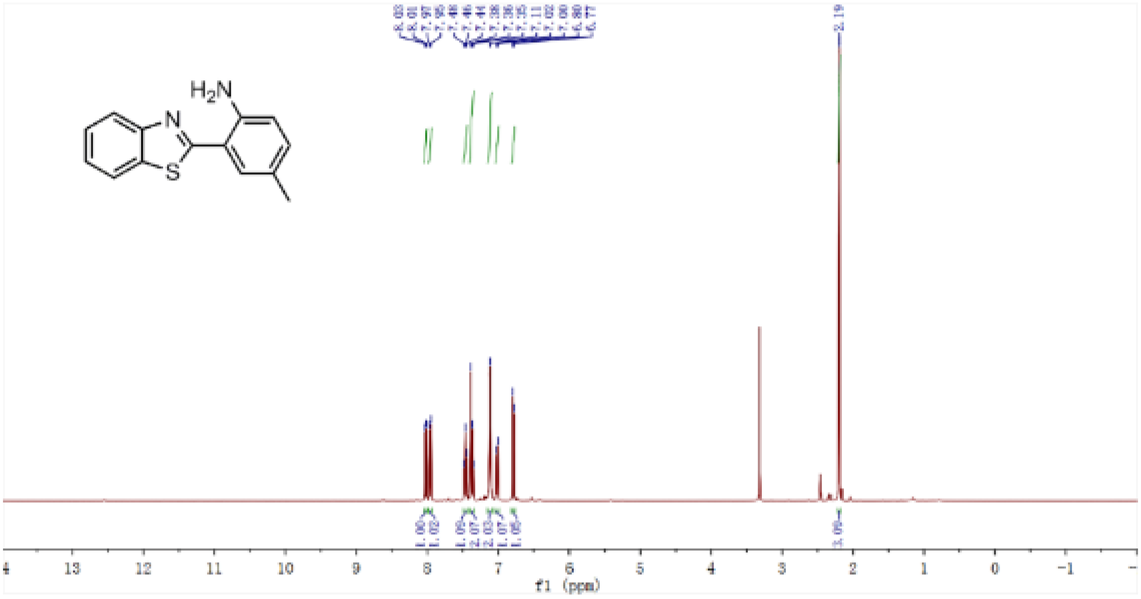

Figure S13. ^{1}H NMR spectra of 2-(2-amino-5-methylphenyl) benzothiazole (DMSO-d_6, 400 MHz).

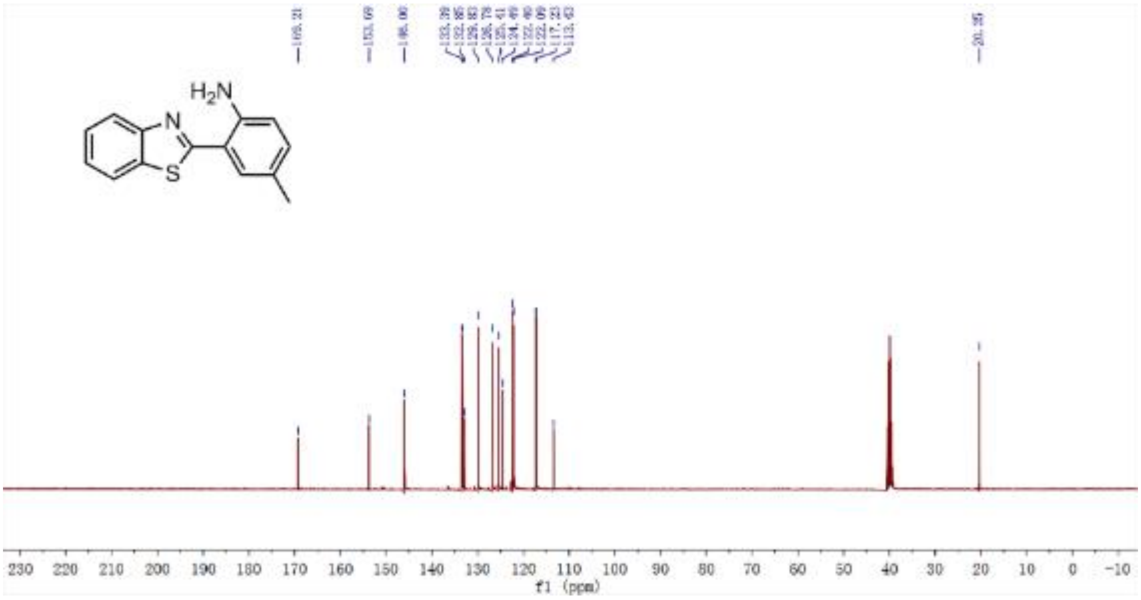

Figure S14. ^{13}C NMR spectra of 2-(2-amino-5-methylphenyl) benzothiazole (DMSO-d_6, 101 MHz).

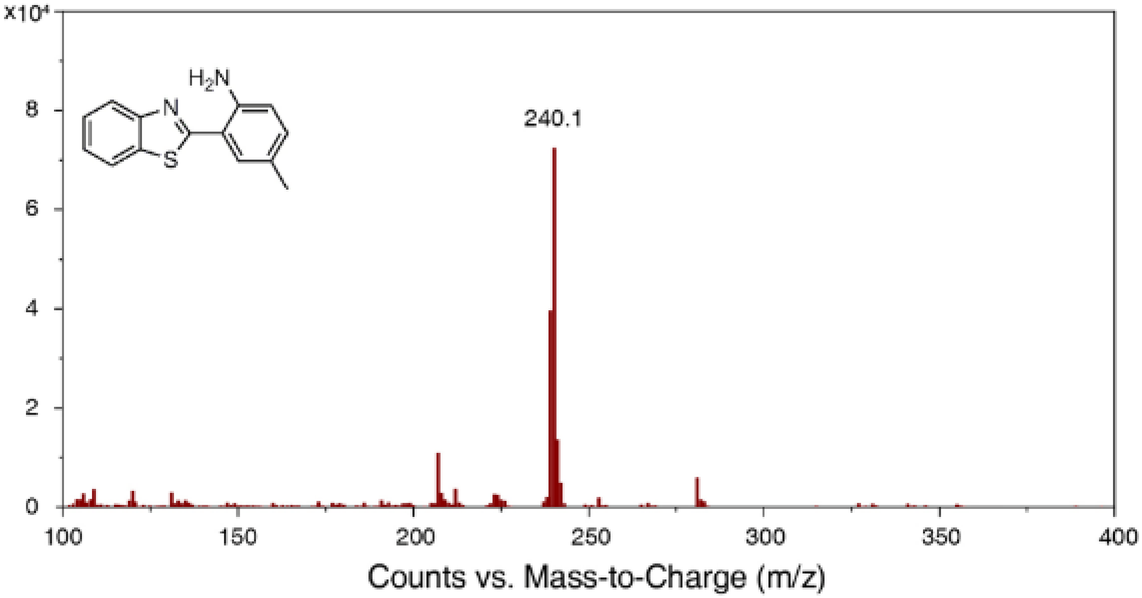

Figure S15. EI spectra of 2-(2-amino-5-methylphenyl) benzothiazole.

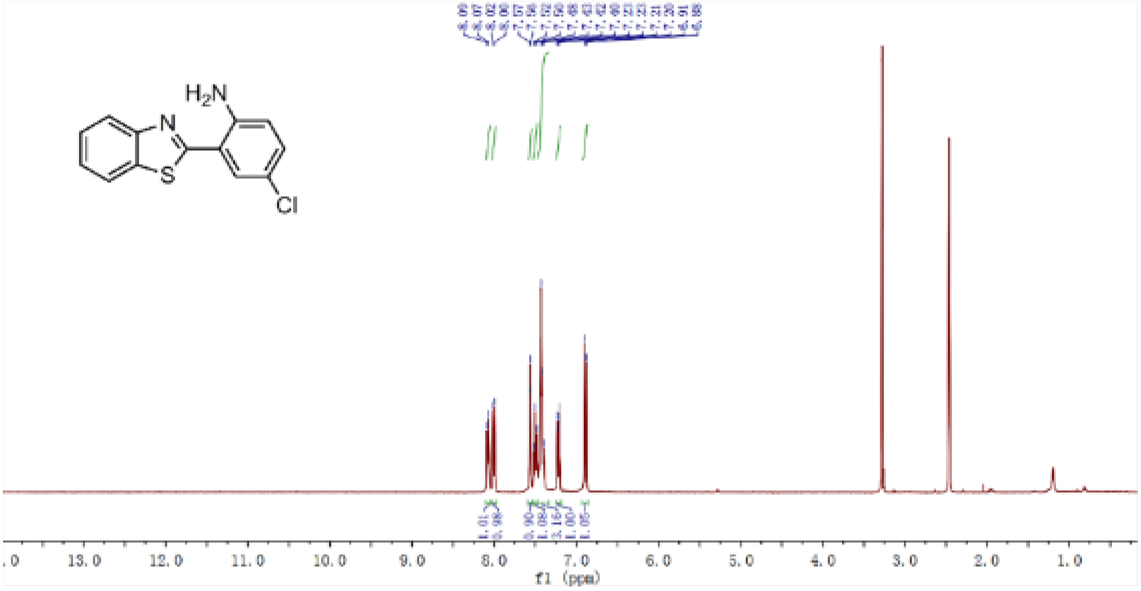

Figure S16. ^{1}H NMR spectra of 2-(2-amino-5-chlorophenyl) benzothiazole (DMSO-d_6, 400 MHz).

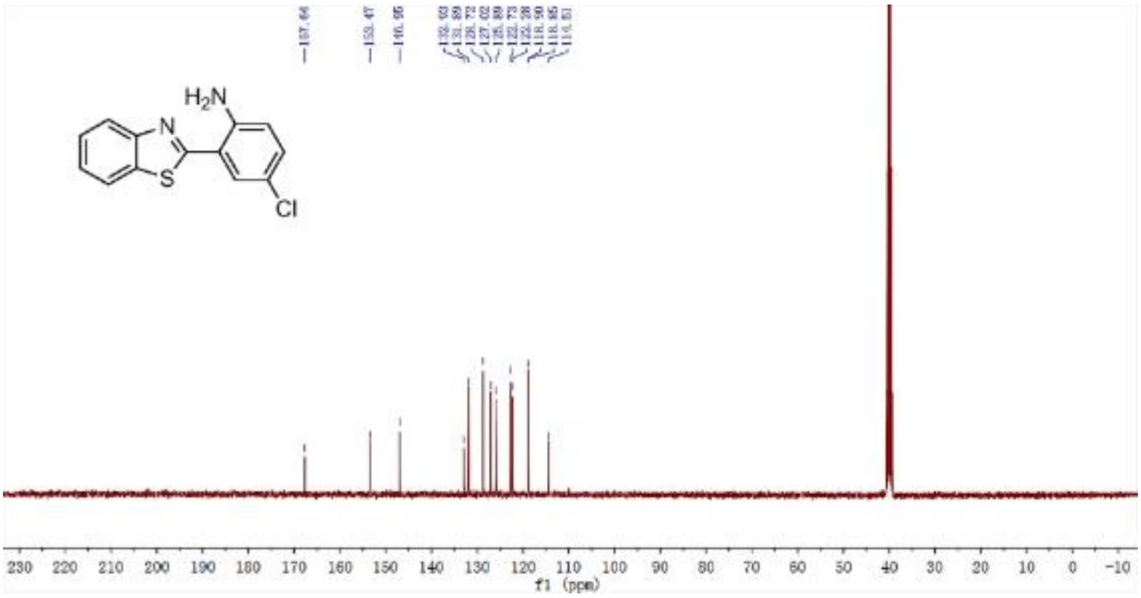

Figure S17. ^{13}C NMR spectra of 2-(2-amino-5-chlorophenyl) benzothiazole (DMSO-d_6, 101 MHz).

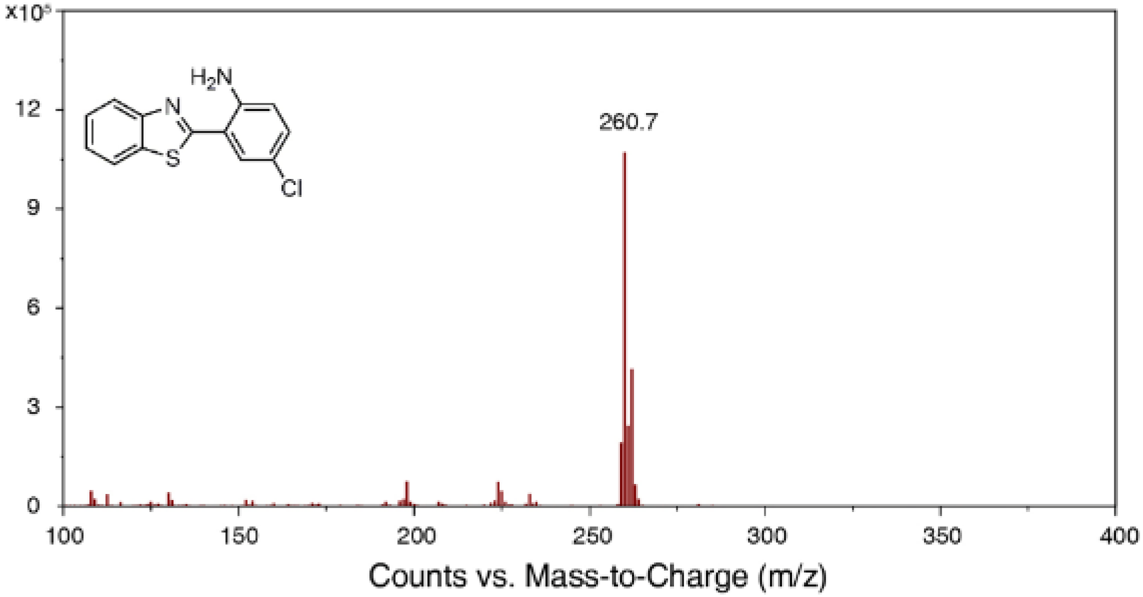

Figure S18. EI spectra of 2-(2-amino-5-chlorophenyl) benzothiazole.

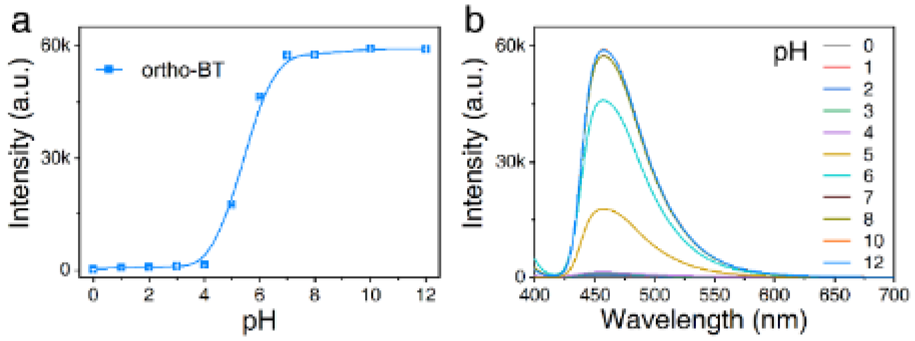

Figure S19. (a) Fluorescence intensity and (b) spectral changes of ortho-BT at pH from 0-12.

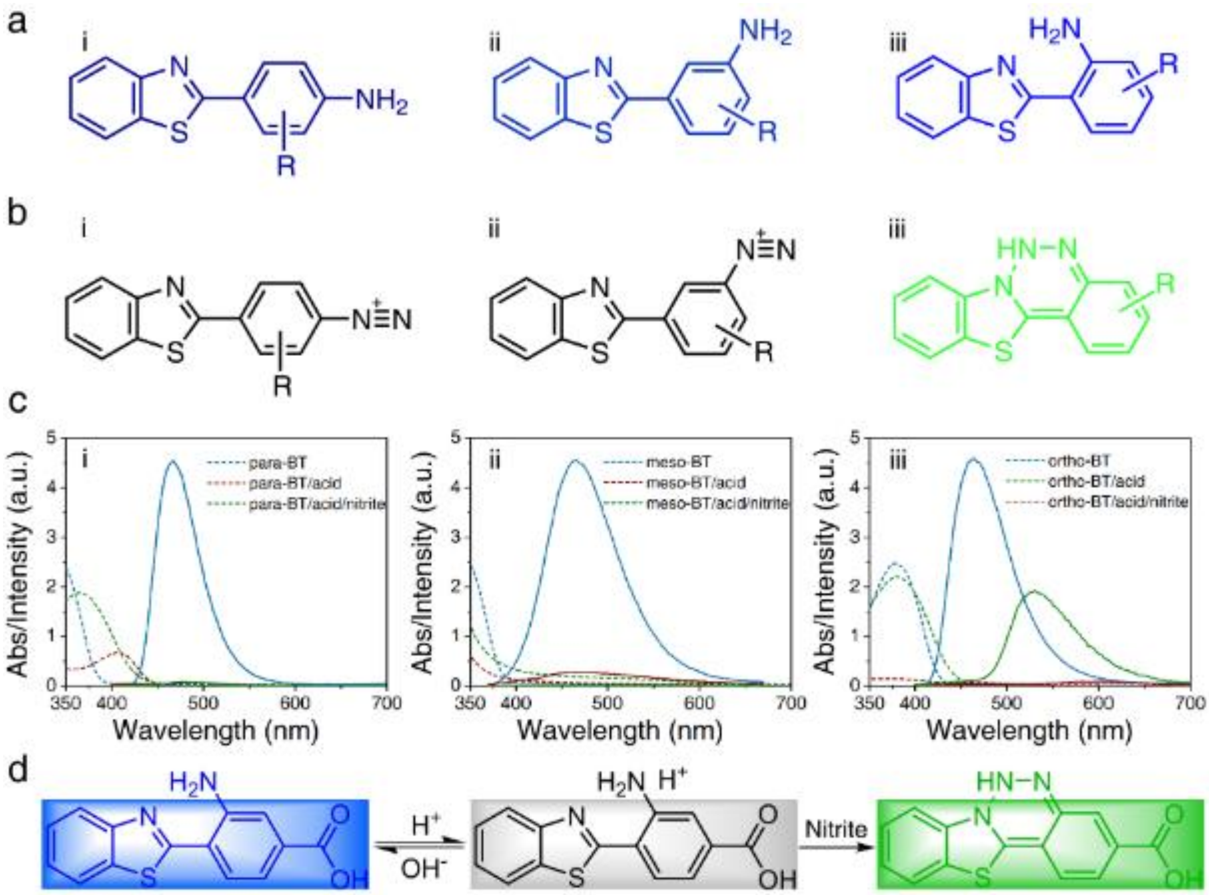

Figure S20. (a) The probes' molecular structure with the binding sites of benzothiazole in the para, meso and ortho sites of aniline, respectively. (b) The corresponding detection mechanism towards nitrite by these three probes. (c) The corresponding spectral responses of the probes to nitrite under acid condition. (d) Proposed detection mechanism of ortho-BT toward nitrite.

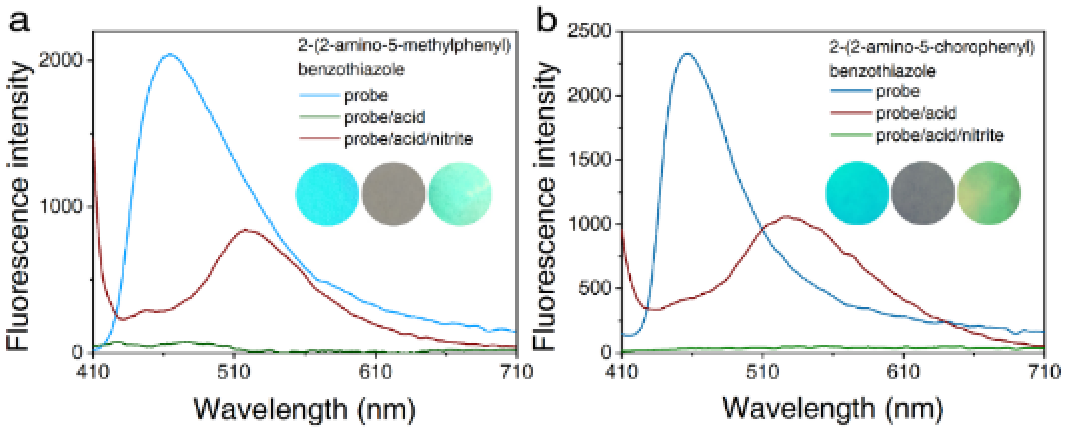

Figure S21. Fluorescence spectral responses of (a) 2-(2-amino-5-chlorophenyl) benzothiazole, and (b) 2-(2-amino-5-methylphenyl) benzothiazole on a paper substrate, with the addition of acid and a further addition of nitrite, respectively. (Inset: The corresponding optical images under an irradiation of a 365 nm UV lamp).

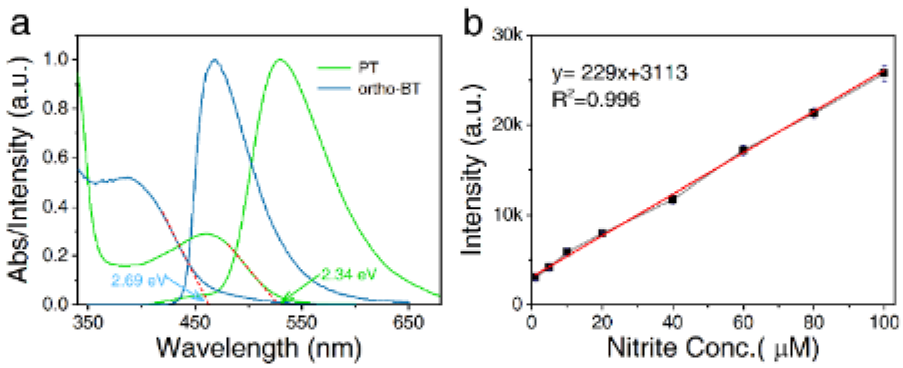

Figure S22. (a) Fluorescence and UV/vis absorbance spectra of ortho-BT and **PT**. (b) The linear fitting of the fluorescence response intensities at 530 nm to various amounts of nitrite (0-100 μM).

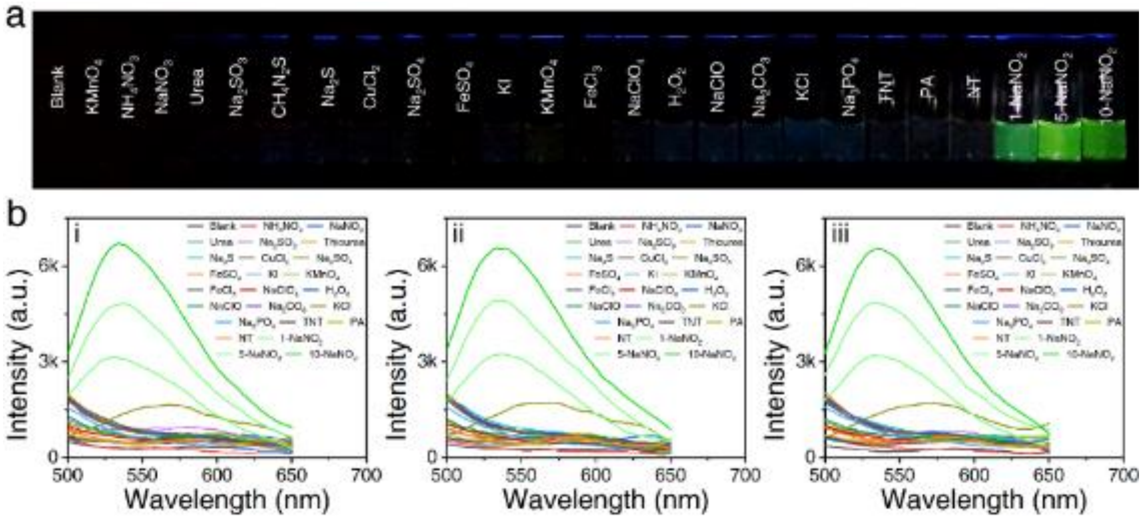

Figure S23. Fluorescence response (a) image, and (b) spectra of ortho-BT (2 mM) in the presence of various test analytes (1 mM) and nitrite (1, 5, 10 μM) solution, respectively (the spectra tested in b

were repeated for three times).

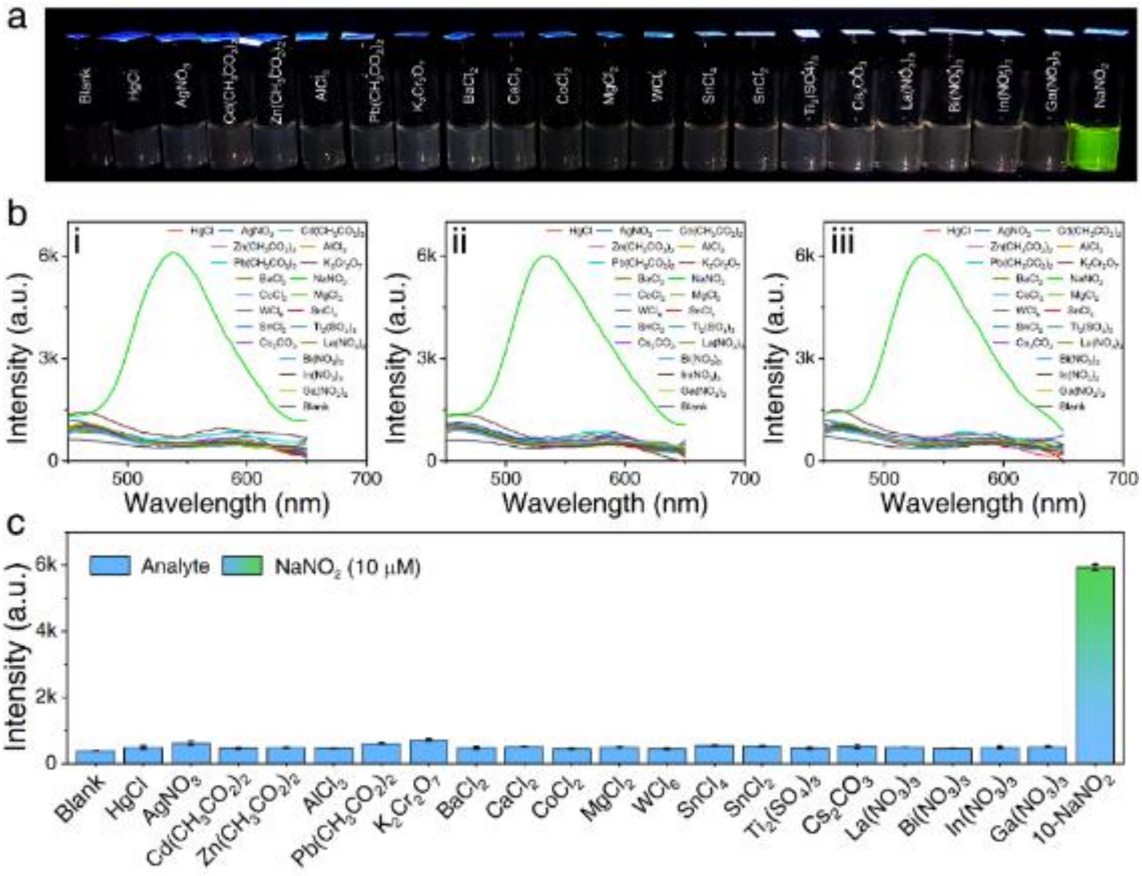

Figure S24. Fluorescence response (a) image, (b) spectra, and (c) intensity changes of ortho-BT (2 mM) in the presence of the various analytes (1 mM) and nitrite (10 μM) solution, respectively (the spectra tested in b were repeated for three times).

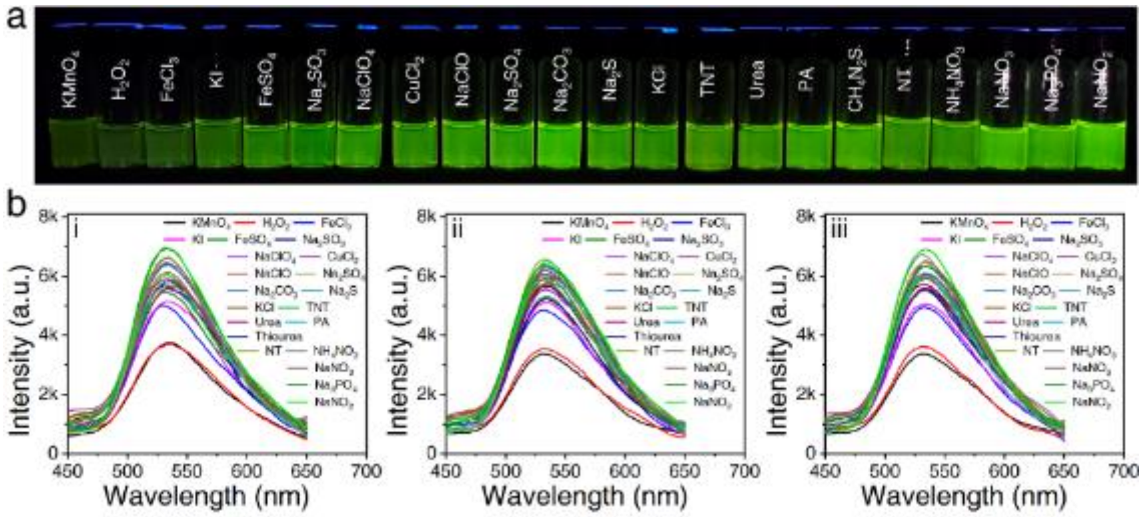

Figure S25. Fluorescence response (a) image, and (b) spectra of ortho-BT (2 mM) in the presence of the mixture solution of various test analytes (1 mM) and nitrite (10 µM), respectively (the spectra tested in b were repeated for three times).

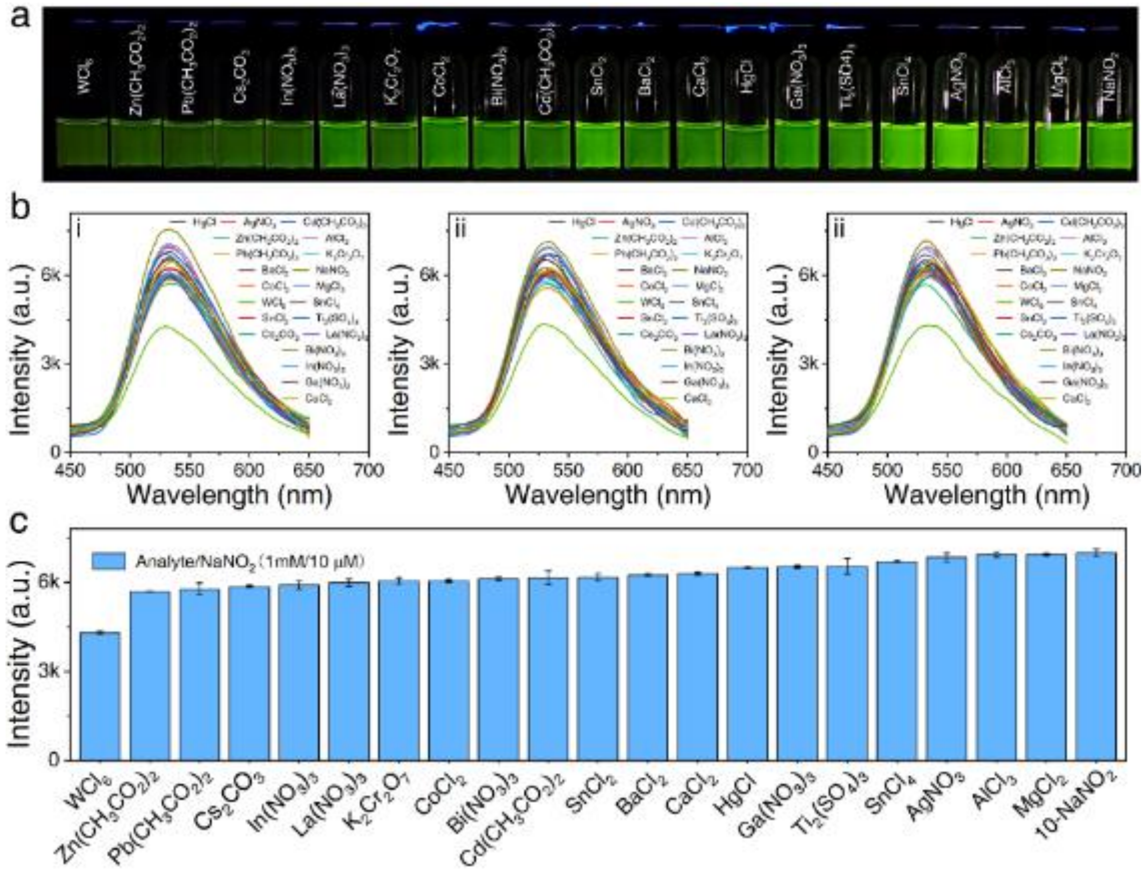

Figure S26. Fluorescence response (a) image, (b) spectra, and (c) intensity changes of ortho-BT (2 mM) in the presence of the mixture solution of various test analytes (1 mM) and nitrite (10 µM), respectively (the spectra tested in b were repeated for three times).

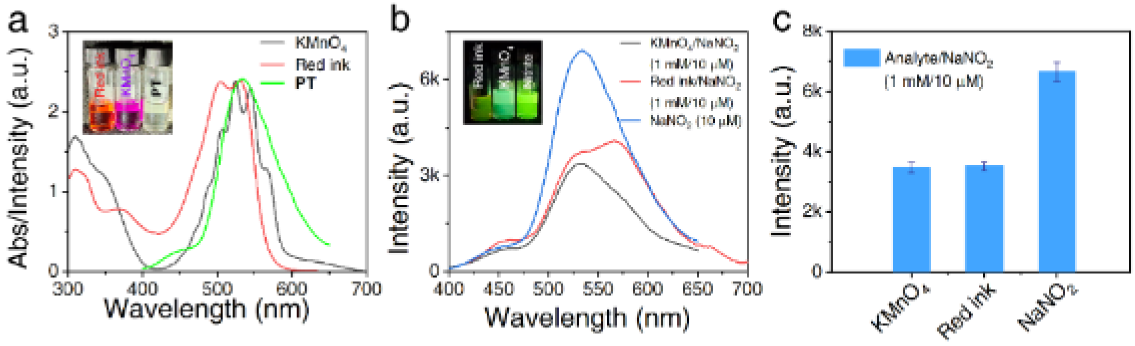

Figure S27. (a) The UV–vis absorption spectra of $KMnO_4$ and red ink overlap with fluorescence spectra of **PT**. Fluorescence (b) spectra and (c) intensity changes obtained for ortho-BT in response to a mixture of nitrite (10 μM) with $KMnO_4$ (1 mM) and red ink, respectively.

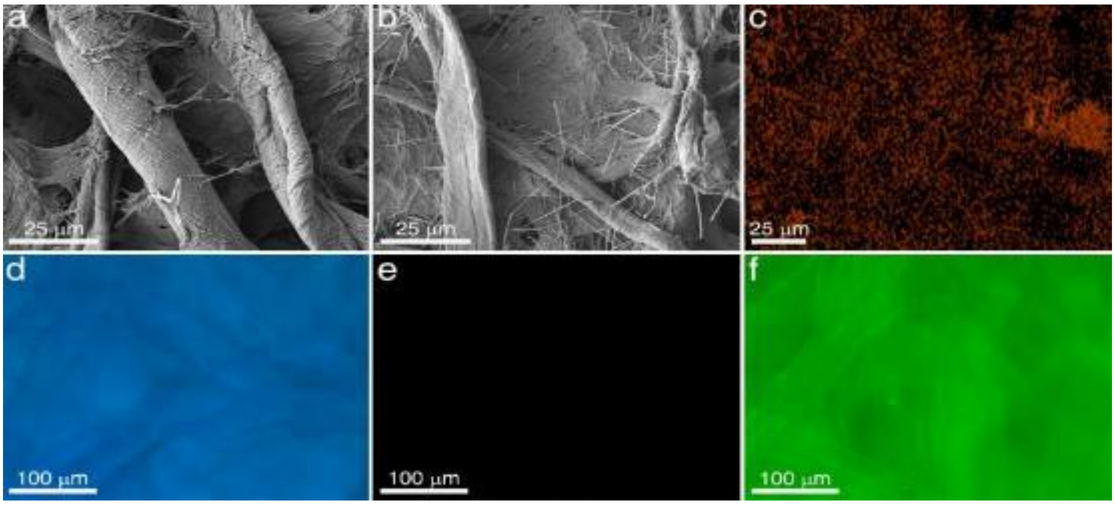

Figure S28. (a) The microstructure of the filter paper. (b) The microstructure of the filter paper with ortho-BT. (c) The corresponding EDS mapping of sulfur (S) element.

Dark field fluorescence images of (d) ortho-BT embedded in the filter paper, (e) before and (f) after the detection of nitrite by the test strip.

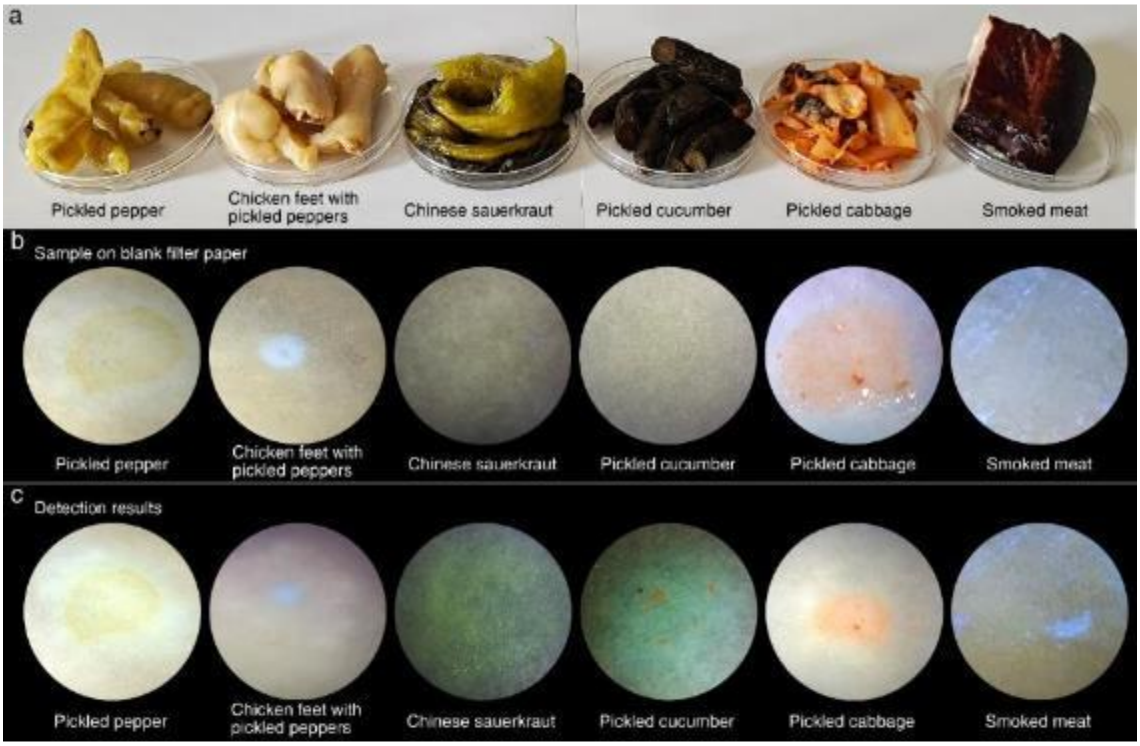

Figure S29. (a) Pickled pepper, chicken feet with pickled pepper, Chinese sauerkraut, pickled cucumber, pickled cabbage, and smoked meat were selected as the real samples. Fluorescence images (Excitation source: 365 nm LED) of real sample on (b) blank filter paper and (c) test trips. (The real samples were purchased from local supermarkets).

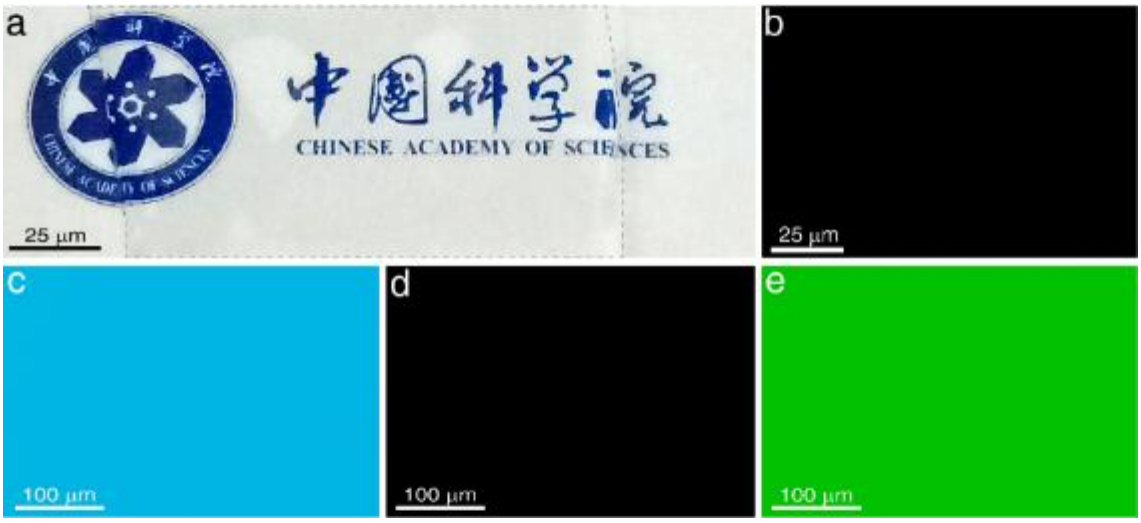

Figure S30. (a) The optical image of the high transmittance of the hydrogel (marked with the dash line area). Dark-field fluorescence images of (b) the hydrogel, (c) the hydrogel with ortho-BT, (d) with a further addition of acid, and (e) a further addition of nitrite.

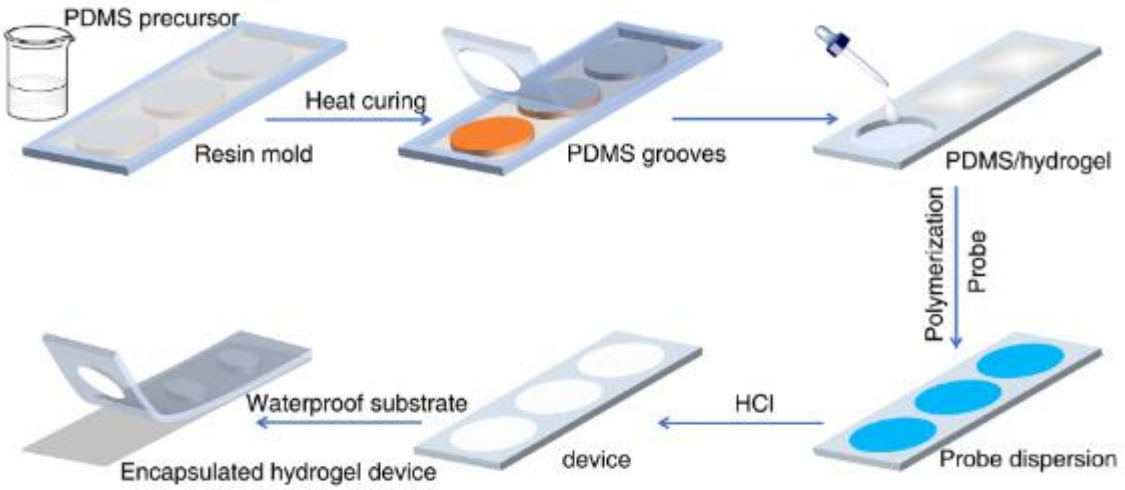

Figure S31. The fabrication processes of the encapsulated hydrogel device.

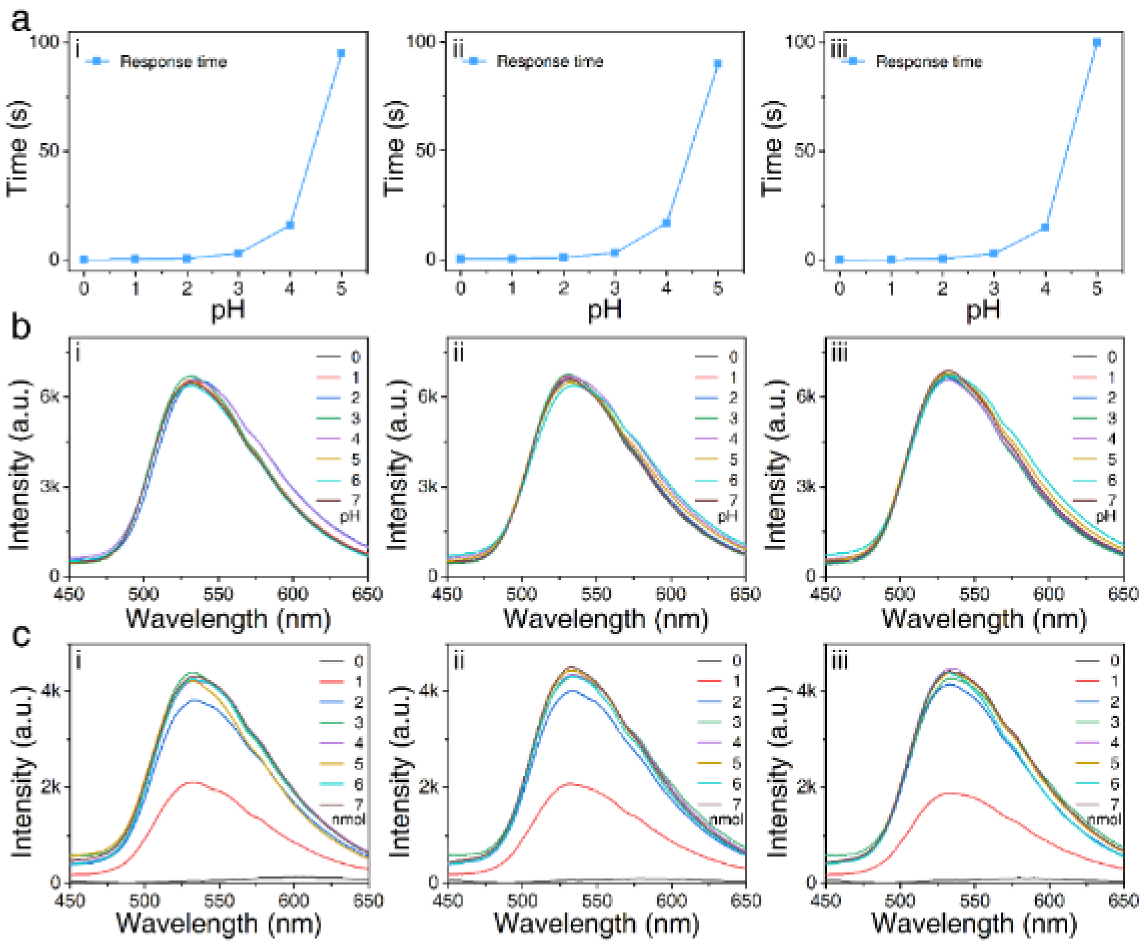

Figure S32. (a) Response time of ortho-BT toward nitrite as a function of pH. The change of fluorescence spectra of the reaction products (**PT**) (b) at pH from 0-7, and (c) with ortho-BT from 0-7 nmol toward nitrite of 2 nmol, respectively (all the spectra tested were repeated for three times). The spectrum in the figure were obtained through the portable fluorescence sensing platform (λ_{ex} = 365 nm; λ_{em} = 530 nm; Slits: 2 nm; Integral time: 100 ms).

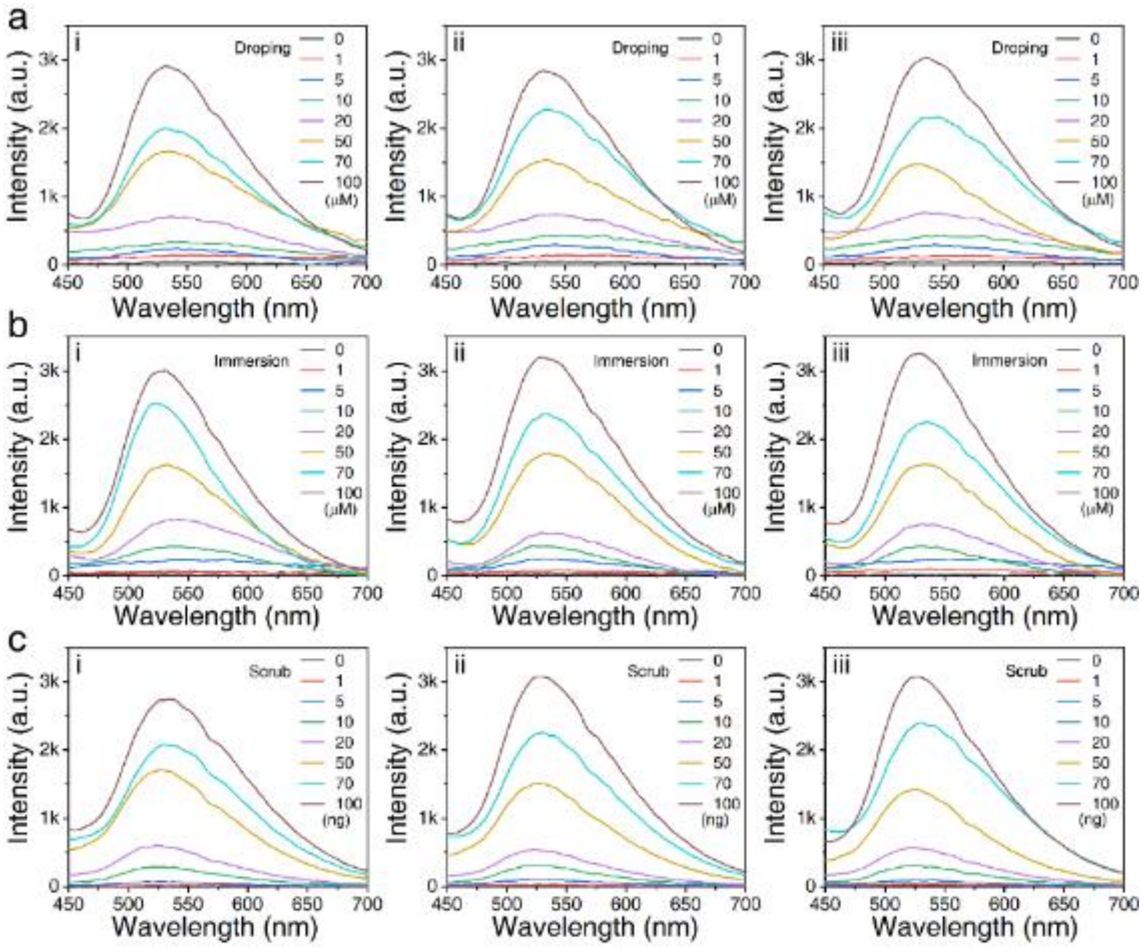

Figure S33. Fluorescence spectra of the test strip upon (a) addition with or (b) immersing into nitrite solution with a concentration of 0–100 μM, respectively. (c) Fluorescence spectra of the encapsulated paper device upon facing with nitrite solid with a mass of 0–100 ng. All the spectra tested were repeated for three times. The spectrum in the figure were obtained through the portable fluorescence sensing platform (λ_{ex} = 365 nm; λ_{em} = 530 nm; Slits: 2 nm; Integral time: 100 ms).

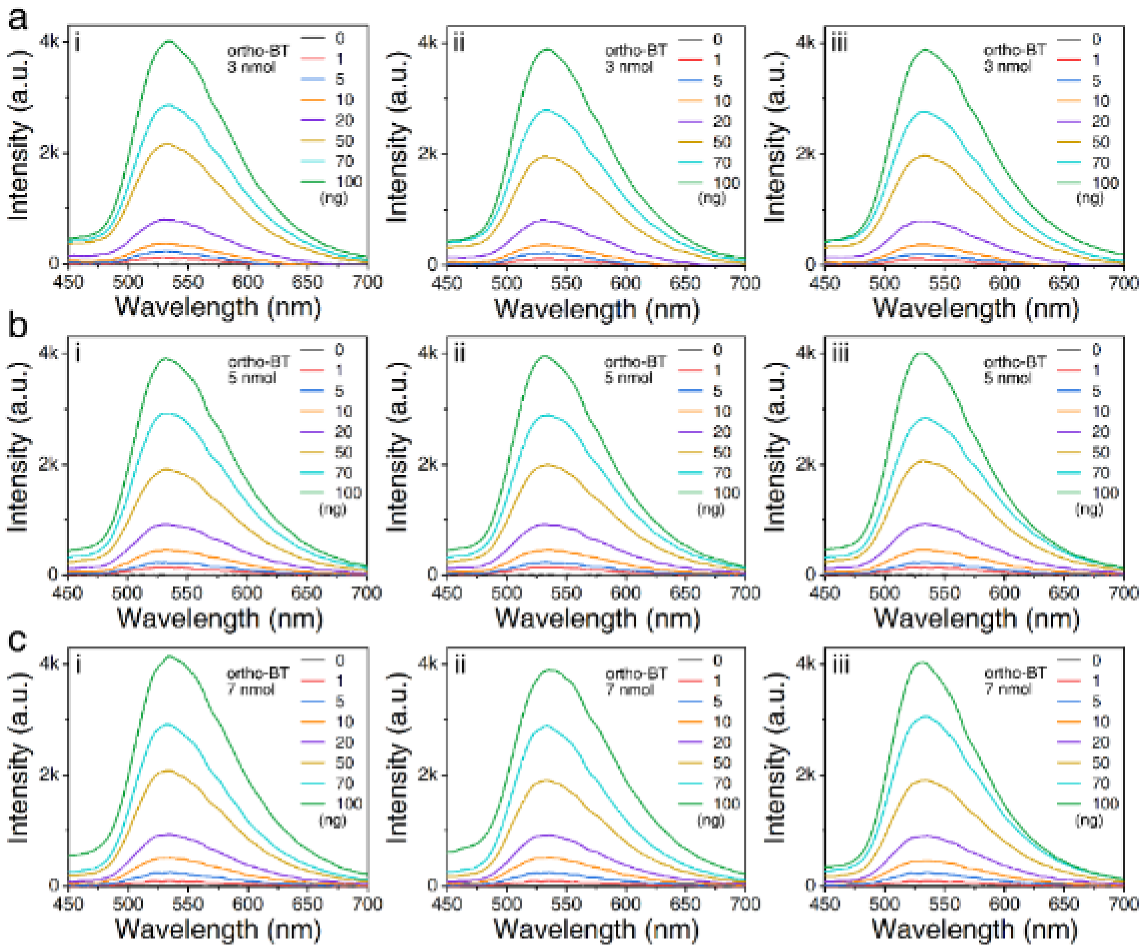

Figure S34. Fluorescence spectra of the encapsulated hydrogel devices containing (a) 3 nmol, (b) 5 nmol, and (c) 7 nmol ortho-BT upon facing with nitrite solid with a mass of 0–100 ng (all the spectra tested were repeated for three times). The spectrum in the figure were obtained through the portable fluorescence sensing platform (λ_{ex} = 365 nm; λ_{em} = 530 nm; Slits: 2 nm; Integral time: 100 ms).

Table S1. Calculated excitation energy (*E*), wavelength (*λ*), and oscillator strength (*f*) for low-laying singlet state (S_n) of ortho-BT and **PT**.[a]

Compound		Main orbital transition (CIC[b])	*E* (eV) [λ (nm)]	*f*
ortho-BT	$S_0 \rightarrow S_1$	HOMO → LUMO (0.70027)	3.1666 eV [391.54 nm]	0.3974
	$S_0 \rightarrow S_2$	HOMO-2 → LUMO (0.17305)<br>HOMO-1 → LUMO (0.66547)	4.0155 eV [308.76 nm]	0.1889
	$S_0 \rightarrow S_3$	HOMO-2 → LUMO (0.66634)<br>HOMO-1 → LUMO (-0.17651)	4.1359 eV [299.77 nm]	0.3109
	$S_0 \rightarrow S_4$	HOMO → LUMO+1 (0.65692)<br>HOMO → LUMO+2 (0.20244)	4.7020 eV [263.68 nm]	0.0657
**PT**	$S_0 \rightarrow S_1$	HOMO → LUMO (0.70290)	0.8476 eV [1462.80 nm]	0.0080
	$S_0 \rightarrow S_2$	HOMO → LUMO+1 (0.66002)<br>HOMO → LUMO+2 (0.24364)	2.3567 eV [526.10 nm]	0.0527
	$S_0 \rightarrow S_3$	HOMO → LUMO+1 (-0.22876)<br>HOMO → LUMO+2 (0.59424)<br>HOMO → LUMO+4 (0.28921)	2.7365 eV [453.08 nm]	0.5463
	$S_0 \rightarrow S_4$	HOMO → LUMO+3 (0.70117)	2.7616 eV [448.96 nm]	0.0002

[a] The optimized structures for the respective models are summarized in theoretical calculations.

[b] CI expansion coefficients for the main transitions.

Table S2. Comparison of the recently developed fluorescent method for the detection of nitrite.

**S. NO.**	Probe	Response time (s)	Linear range (μM)	LOD (nM)	Naked eye LOD (μM)	Ref.
1	Carbon dots	600	0.0625-2	20	50	[4]
2	Carbon nanodots	1200	/	94	/	[5]
3	BODIPY	1800	0.1-8	15	/	[6]
4	Carbon quantum dots	180	0.02-1	3.3	/	[7]
5	2-amino-6-chlorobenzoic acid	6000	2-50	120	/	[8]
6	Quantum dots	/	0.1-100	50	/	[9]
7	Rhodamine	1200	/	1200	/	[10]
8	Carbon nanodots	300	0-1000	1000	/	[11]
9	Lanthanide coordinationpoly	600			/	[12]
10	Phenanthroimidazol	600	0.1-10	43	/	[13]
11	Copper (II) complex	600	/	/		[14]
12	Poly(ophenylenediamine)–Rhodamine B copolymer dots	/	/			[15]
13	Rhodamine B	900	5-100	/	4	[16]
**14**	**2-(2-amino) benzothiazole derivatives**	**< 5**	**1-100**	**40**	**1 (5 ng)**	**This work**

References

[1] J. P. Perdew, K. Burke, M. Ernzerhof, *Phys. Rev. Lett.* **1996**, *77*, 3865-3868.

[2] F. Weigend, R. Ahlrichs, *Phys. Chem. Chem. Phys.* **2005**, *7*, 3297-3305.

[3] T. Lu, F. Chen, *J. Comput. Chem.* **2012**, 33, 580-592.

[4] Y. Zhan, Y. Zeng, L. Li, F. Luo, B. Qiu, Z. Lin, L. Guo, *ACS Sens.* **2019**, *4*, 1252-1260.

[5] B. L. Li, Y. S. Li, X. F. Gao, *Food Chem.* **2019**, *274*, 162-169.

[6] J. Zhang, F. Pan, Y. Jin, N. Wang, J. He, W. Zhang, W. Zhao, *Dyes Pigments* **2018**, *155*, 276-283.

[7] M. Zan, L. Rao, H. Huang, W. Xie, D. Zhu, L. Li, X. Qie, S.-S. Guo, X.-Z. Zhao, W. Liu, W.-F. Dong, *Sens. Actuators B* **2018**, *262*, 555-561.

[8] H. Wang, N. Wan, L. Ma, Z. Wang, B. Cui, W. Han, Y. Chen, *Analyst* **2018**, *143*, 4555-4558.

[9] H. H. Ren, Y. Fan, B. Wang, L. P. Yu, *J. Agric. Food Chem.* **2018**, *66*, 8851-8858.

[10] Y. Zhao, H. Wang, G. Xu, L. Li, L. Liu, *Sens. Actuators B* **2017**, *246*, 783-791.

[11] H. Zhang, S. Kang, G. Wang, Y. Zhang, H. Zhao, *ACS Sens.* **2016**, *1*, 875-881.

[12] Z. Qi, Q. You, Y. Chen, *Anal. Chim. Acta* **2016**, *902*, 168-173.

[13] B. Gu, L. Huang, J. Hu, J. Liu, W. Su, X. Duan, H. Li, S. Yao, *Talanta* **2016**, *152*, 155-161.

[14] K. C. Rout, B. Mondal, *Inorg. Chim. Acta* **2015**, *437*, 54-58.

[15] F. Liao, X. Song, S. Yang, C. Hu, L. He, S. Yan, G. Ding, *J. Mater. Chem. A* **2015**, *3*, 7568-7574.

[16] Z. Xue, Z. Wu, S. Han, *Anal. Methods* **2012**, *4*, 2021-2026.
